# Effects of α-Synuclein on the Lipid Phenotype of SZ95 Human Sebocytes: A Preliminary Study in the Context of Parkinson’s Disease

**DOI:** 10.3390/cells15161505

**Published:** 2026-08-21

**Authors:** Sarah Mosca, Grazia Bottillo, Enrica Flori, Daniela Kovacs, Miriam Maiellaro, Francesca Lozzi, Alessia Cavallo, Christos C. Zouboulis, Giulia Simmini, Alessia Luppino, Claudia Novello, Valentina Leta, Gianfranco Gaudiano, Grazia Devigili, Roberto Eleopra, Fabrizio Tagliavini, Samanta Mazzetti, Emanuela Camera, Giorgia Cardinali

**Affiliations:** 1 Laboratory of Cutaneous Physiopathology and Integrated Center of Metabolomics Research, San Gallicano Dermatological Institute, IRCCS, 00144 Rome, Italy; sarah.mosca@ifo.it (S.M.); grazia.bottillo@ifo.it (G.B.); daniela.kovacs@ifo.it (D.K.); miriam.maiellaro@ifo.it (M.M.); francesca.lozzi@ifo.it (F.L.); alessia.cavallo@ifo.it (A.C.); emanuela.camera@ifo.it (E.C.); giorgia.cardinali@ifo.it (G.C.); 2 Department of Dermatology, Venereology and Immunology, Faculty of Health Sciences, Brandenburg Medical School Theodor Fontane, Universitaetsklinikum Ruppin-Brandenburg, 16816 Neuruppin, Germany; christos.zouboulis@mhb-fontane.de; 3 Department of Skin and Venereal Diseases, Lithuanian University of Health Sciences, LT-44307 Kaunas, Lithuania; 4 Dementia and Degenerative Diseases of CNS Unit, Fondazione IRCCS Istituto Neurologico Carlo Besta, 20133 Milan, Italy; giulia.simmini@istituto-besta.it; 5 Parkinson’s and Movement Disorders Unit, Department of Clinical Neurosciences, Fondazione IRCCS Istituto Neurologico Carlo Besta, 20133 Milan, Italy; alessia.luppino@istituto-besta.it (A.L.); claudia.novello@istituto-besta.it (C.N.); valentina.leta@istituto-besta.it (V.L.); gianfranco.gaudiano@istituto-besta.it (G.G.); grazia.devigili@istituto-besta.it (G.D.); roberto.eleopra@istituto-besta.it (R.E.); samanta.mazzetti@istituto-besta.it (S.M.); 6 Department Medical Biotechnology and Translational Medicine, Università Degli Studi di Milano, 20122 Milan, Italy; 7 Fondazione IRCCS Istituto Neurologico Carlo Besta, 20133 Milan, Italy; fabrizio.tagliavini@istituto-besta.it

**Keywords:** Parkinson’s disease, neurodegeneration, seborrheic dermatitis, α-synuclein, sebocyte, sebaceous gland

## Abstract

**Highlights:**

**What are the main findings?**

- Oligomeric α-synuclein promotes differentiation of human SZ95 sebocytes.
- α-Synuclein alters lipogenic pathways and remodels SZ95 sebocytes lipid composition.

**What are the implications of the main findings?**

- These findings provide preliminary evidence for α-synuclein involvement in sebocyte biology.
- Sebocyte lipid remodeling may represent a peripheral response to pathological α-synuclein.
- The findings support further investigation of sebaceous glands as peripheral targets of α-synuclein pathology.

**Abstract:**

Pathological aggregation of α-synuclein (αSyn) is a hallmark of Parkinson’s disease (PD), but increasing evidence indicates that αSyn deposits extend beyond the central nervous system to peripheral tissues, including the skin. In PD patients, cutaneous αSyn accumulation has been associated with seborrheic dermatitis (SD), a chronic inflammatory condition linked to sebocyte dysfunction and altered sebum production. Marked differences in sebum composition between PD patients and healthy controls have been described. We aimed to investigate whether pathological αSyn could contribute to dysregulated sebum production and composition by assessing key markers of sebocyte differentiation and lipogenesis in human sebaceous gland cells and skin biopsies from PD patients (*n* = 5) and matched controls (*n* = 5) without clinically recorded SD. O-αSyn exposure of immortalized sebaceous gland cells (SZ95) altered the transcriptional programs related to sebocyte differentiation, inflammation, metabolism, and lipogenesis. Consistently, protein markers of mid-to-late sebocyte differentiation were increased. Upregulation of PLIN2, together with elevated PPARγ, SREBP1, SCD1, and FADS2, indicated progression toward a mature and lipid-producing phenotype characterized by enhanced accumulation of neutral lipids within lipid droplets. Lipidomic profiling revealed remodeling of multiple lipid classes, with increases in triglycerides, ceramides, and selected phospholipids. Increased expression of PLIN2 and PPARγ was also observed in sebaceous glands from PD skin biopsies compared to controls. Collectively, our findings indicate that O-αSyn exposure is associated with molecular changes consistent with altered sebocyte differentiation and lipid remodeling. These results support a potential association between αSyn and lipid dysregulation in sebocytes and suggest that these cells may represent unrecognized peripheral targets of αSyn.

## 1. Introduction

Alpha-synuclein (αSyn) is a presynaptic protein implicated in neurodegenerative diseases, most notably Parkinson’s disease (PD), a multisystem disorder characterized by progressive neuronal degeneration, resulting in a gradual decline in autonomic, motor and cognitive functions. The αSyn misfolding and aggregation into oligomeric and fibrillar forms lead to Lewy body formation and dopaminergic neuron loss in PD patients [1,2,3,4,5]. Increasing evidence suggests that oligomeric and fibrillar αSyn aggregates are not only present in the brain but also in peripheral tissues, including the skin, where higher amounts of αSyn deposits have been detected in PD patients [6,7,8]. Elevated levels of αSyn have been observed in keratinocytes of the spinous layer, fibroblasts, and in the pilosebaceous unit of patients with PD [9,10]. Notably, αSyn deposits are present in over 60% of cells within the pilosebaceous unit. Cutaneous accumulation of αSyn is detectable even at early stages of PD, indicating that αSyn begins to accumulate during the premotor phase [11].

The deposition of αSyn in the skin of PD patients has been associated with an increased incidence of seborrheic dermatitis (SD), a chronic inflammatory skin disorder affecting sebum-rich areas (e.g., scalp, face, and upper trunk) characterized by hyperseborrhea and desquamation [12,13,14,15,16]. SD affects approximately 52–59% of PD patients, with male predominance [17,18] and is linked to dysregulation of sebocytes, the specialized epithelial cells responsible for sebum production, as well as the shaping of microbial growth and immune responses. The pathogenesis of SD involves a complex interplay of factors beginning with overactive sebaceous glands (SGs) that secrete excessive amounts of sebum with altered composition [19]. The dysregulated SGs product sets up an environment favorable to the proliferation of Malassezia yeasts, which release lipases and phospholipases that hydrolyze sebum triglycerides (TGs) into irritating fatty acids and lipid peroxides. These byproducts further promote fungal outgrowth and trigger inflammation. The immune response, through cytokines and complement activation, induces keratinocyte proliferation, ceramide loss with disruption of the skin barrier, and stratum corneum thickening, ultimately leading to the characteristic SD symptoms: erythema, itching, and scaling [14,15,20,21].

The complex interactions between the central nervous system and the skin are not yet fully understood, and the exact mechanisms underlying the bidirectional skin–brain axis remain unclear. The emerging link between PD and SD raises the intriguing possibility that pathological αSyn deposits may accumulate beyond the central nervous system, such as in the skin, potentially contributing to cutaneous manifestations. In this context, SD may represent an early sign of PD occurring before motor deficits manifest. When associated with PD, SD is often particularly severe and resistant to conventional treatments [22,23,24]. Dysregulation of the hypothalamic–pituitary–adrenal (HPA) axis has been described in PD, and αSyn accumulation in the skin may interfere with SG function, leading to altered sebum secretion and composition. Interestingly, recent studies have revealed profound differences in sebum composition between patients with PD and healthy controls [25,26,27,28,29]. This study aims to investigate the possible link between αSyn and sebocyte lipid dysregulation by evaluating characteristic markers of sebocyte differentiation and function in the SZ95 sebocyte cell line exposed to αSyn oligomers (O-αSyn), and in the SGs of skin biopsies obtained from PD patients (*n* = 5) compared to control subjects (*n* = 5).

## 2. Materials and Methods

### 2.1. Materials

Sebomed^®^ basal medium was obtained from Merck-Biochrom (Berlin, Germany). Fetal bovine serum (FBS) was purchased from Cytiva Hyclone (Milan, Italy). L-glutamine (2 mM), penicillin (100 U/mL), streptomycin (100 µg/mL), trypsin/EDTA, recombinant human epidermal growth factor, and D-PBS were purchased from Invitrogen Technologies (Monza, Italy). Human Recombinant Alpha-Synuclein Oligomers (O-αSyn) (kinetically stable) were obtained from StressMarq Biosciences (Cat. No. SPR-484) (Victoria, BC, Canada). According to the manufacturer’s specifications, the preparation consists of recombinant human α-synuclein oligomers with >95% purity (Native-PAGE), structurally characterized by transmission electron microscopy (TEM), and retaining approximately 90% of the oligomeric species after 14 days at 37 °C and after a freeze–thaw cycle. AurumTM Total RNA mini kit and Bradford reagent were purchased from Bio-Rad (Milan, Italy). First Strand cDNA synthesis kit was from Diatech Labline (Jesi, Italy). SYBR Green PCR Master Mix was purchased from Neobiotech (Nanterre, France). GAPDH antibody (G9545) was from Sigma-Aldrich (Milan, Italy). The antibodies to K7 (#4465), PPARγ (D9 #2430), BLIMP1 (#9115), a secondary anti-mouse IgG HRP-conjugated antibody and anti-rabbit IgG HRP-conjugated antibody were purchased from Cell Signaling (Danvers, MA, USA); Anti-EMA antibody (ab156947) and anti-FADS2 antibody (ab72189) were purchased from Abcam (Abcam, Cambridge, UK). Anti-SREBP1 (sc-13551) and anti-PPARγ (sc-7273) antibodies were purchased from Santa Cruz Biotechnology (1422 DR, Uithoorn, The Netherlands). Anti-alpha-synuclein monoclonal antibody (Syn211) (#AHB0261) and anti-PLIN2/ADFP (#MA5-38601) mouse monoclonal antibody were purchased from Invitrogen, Thermo Fisher Scientific. Amersharm ECL Western Blotting Detection Reagent was from GE Healthcare (Buckinghamshine, UK). RIPA lysis buffer, broad spectrum protease inhibitor cocktail, and broad-spectrum phosphatase inhibitor cocktail were from Boster Biological Technology Co. (Pleasanton, CA, USA).

### 2.2. Chemicals

HPLCMS-grade acetone, ethyl acetate, and chloroform were purchased from Carlo Erba (Milan, Italy). HPLCMS-grade acetonitrile, isopropanol, and methanol were purchased from Biosolve (Chimie SARL, Dieuze, France; BV, Valkenswaard, The Netherlands), while ultra-HPLCMS-grade water was purchased from LiChrosolv by Merck (Darmstadt, Germany). Butylated hydroxytoluene (BHT) and the mobile-phase modifiers ammonium formate (NH4COOH) and ammonium fluoride (NH4F) were purchased from Sigma Aldrich (Milan, Italy). Deuterium-labeled ceramide LIPIDOMIX^®^ Mass Spec Standard Solution, EquiSPLASH™ LIPIDOMIX^®^ Mass Spec Standard Solution, and N-palmitoyl-d31-D-erythro-sphingosine (d31-Cer16:0, MW 569.1) were purchased from Avanti Polar Lipids (Alabaster, AL, USA). Deuterated cholesterol-2,2,3,4,4,6-d6 (d6-CH, MW 392), deuterated cholesterol sulfate sodium salt (d7-CHS, MW 495), and hexadecanoic-9,9,10,10,11,11,12,12,13,13,14,14,15,15,16,16,16d17 acid (d17-PA, MW 273), glyceryl trihexadecanoate-d98 (d98TG 48:0, MW 906), and n-hexadecyl-1,1,2,2-d4 hexadecanoate-16,16,16-d3 (d7WE, MW 488) were purchased from CDN Isotopes Inc. (Pointe-Claire, QC, Canada). Details on the internal standards (iSTDs) used are reported in Supplementary Table S1. The mass calibration solution was prepared from Agilent Technologies Tuning mix (HP0321 solution, Agilent Technologies, Santa Clara, CA, USA) upon dilution in acetonitrile.

### 2.3. Study Design

The objectives of this study were (i) to assess key markers of sebocyte differentiation, inflammation and metabolism following exposure to O-αSyn; (ii) to explore possible dysregulation in sebum production and composition; and (iii) to evaluate the expression of lipid droplet-associated proteins in skin biopsies from PD patients and matched controls. This study was carried out on patients evaluated at the Movement Disorder Unit of the Neurological Institute “Carlo Besta” (Milan, Italy) by neurologists experienced in the movement disorders field. The study was approved by the ethical committee (ID 6068 RF-2021-12375222; PNRR-MAD-2022-12376035). The study was performed in agreement with the principles and guidelines of the Declaration of Helsinki. Immortalized human sebaceous gland cells (SZ95) were treated with O-αSyn for 24 h or 1 week. Skin biopsies were collected from 5 PD patients and 5 age-matched healthy controls. Expression of genes related to differentiation, inflammation, and lipid synthesis was evaluated by array card analysis and RT-PCR. Sebocyte differentiation and lipogenesis protein markers were assessed in SZ95 cells and biopsies by Western blot and immunofluorescence. Lipidomic analysis of SZ95 cells was performed using GCMS and LCMS. For each experiment, at least three biological replicates were used, and data analyses were not blinded. Details of *p*-values are reported in each figure legend.

### 2.4. Cell Cultures and Stimulation with O-αSyn

The immortalized human sebocyte line SZ95, which recapitulates the morphological, phenotypic, and functional features of normal human sebocytes, was kindly provided by Prof. Zouboulis [30]. Cells were maintained in Sebomed^®^ basal medium supplemented with 10% FBS, 2 mM L-glutamine, 100 µg/mL penicillin/streptomycin, 5 ng/mL recombinant human epidermal growth factor, and 1 mM CaCl_2_. Cultures were incubated at 37 °C in a humidified atmosphere containing 5% CO_2_ and were routinely monitored for mycoplasma contamination. For routine maintenance, cells were subcultured at approximately 70–90% confluence. For experimental treatments, SZ95 sebocytes were exposed to O-αSyn at concentrations of 2, 5, or 10 µg/mL, with the culture medium replaced every other day. Cells were collected at 24 h and 7 days following stimulation for subsequent assessment of gene and protein expression and lipid profiles.

### 2.5. Skin Biopsies

This study was conducted in accordance with the Declaration of Helsinki, including written informed consent to the use of anonymized patient clinical data for research purposes according to local regulatory requirements. The study was approved by the ethical committee (ID 6068 RF-2021-12375222; PNRR-MAD-2022-12376035). The subjects were evaluated at the Movement Disorder Unit of the Neurological Institute “Carlo Besta” (Milan, Italy) by neurologists experienced in the movement disorders field, who collected demographic and clinical information.

The selected patients were affected only by PD in order to exclude the involvement of dermatological issues (comorbidities and topical treatments). Skin biopsies (*n* = 10 male subjects: 5 PD patients and 5 age-matched healthy controls; Table 1) were processed following the standard procedure [31]. Briefly, after local anesthesia, a 3 mm punch biopsy was taken from the cervical C7 paravertebral area, and the skin samples were immediately fixed in Zamboni’s fixative and kept at 4 °C for 5 h. After cryoprotection, skin biopsies were cut using a cryostat into fifty-micron-thick free-floating sections.

SD and dermatological comorbidities were recorded from clinical history and dermatological examination. No topical treatments with antifungal, corticosteroid or cosmetic preparations had been applied to the sampling area within the 2 weeks preceding biopsy.

### 2.6. Direct Total Cell Counts by Flow Cytometry

Following exposure to O-αSyn (2, 5, or 10 μg/mL) for 24, 48, or 72 h, SZ95 sebocytes were harvested by trypsinization and resuspended as a single-cell suspension in 500 μL PBS containing 0.1% FBS. For flow cytometric analysis, 100 μL of each sample was analyzed in technical duplicate using a MACSQuant Analyzer 10 flow cytometer (Miltenyi Biotec, Milan, Italy). To evaluate potential O-αSyn-induced cytotoxicity, cell viability was assessed in parallel by the trypan blue exclusion method. Cells were counted using an Axiovert 40C phase-contrast microscope (Zeiss, Milan, Italy). None of the concentrations tested resulted in detectable trypan blue positivity. Results are reported as mean ± SD from three biological replicates.

### 2.7. Flow Cytometric Analysis of Cell Cycle

Following treatment, SZ95 sebocytes were harvested by trypsinization and fixed overnight at 4 °C in ice-cold 70% ethanol to preserve cellular DNA for subsequent analysis. Fixed cells were washed and stained with a PBS-based solution containing propidium iodide (50 μg/mL) and RNase A (40 μg/mL). DNA content was subsequently quantified by flow cytometry using a MACSQuant Analyzer. For each sample, 10^4^ events were collected, with all measurements performed in technical duplicate. Flow cytometric profiles were evaluated by identifying the pre-G1, G1, S, and G2/M cell cycle populations. Data analysis was performed using MACSQuantify 2.13 Software. Results are expressed as mean ± SD from three biological experiments.

### 2.8. RNA Extraction, Gene Expression Array Card Analysis, and Quantitative Real-Time RT-PCR

Total RNA was extracted with the Aurum™ Total RNA Mini Kit (Bio-Rad Laboratories, Inc., Hercules, CA, USA) following the manufacturer’s protocol and stored at −80 °C until further processing. After DNase I treatment, reverse transcription was performed using the PrimeScript™ RT Master Mix cDNA synthesis kit (Takara Bio USA, Inc., San Jose, CA, USA), according to the manufacturer’s instructions. cDNA samples were loaded onto 384-well microfluidic cards configured with TaqMan probe/primer sets targeting 93 genes of interest and three reference genes (18S rRNA, GAPDH, and β-actin). Real-time PCR was carried out using an Applied Biosystems^®^ QuantStudio™ 7 Flex instrument (Thermo Fisher Scientific, Monza, Italy), and data were processed with QuantStudio™ Design and Analysis Software v2 (Thermo Fisher Scientific). For targeted gene-expression analysis, real-time RT-qPCR reactions were performed in a final volume of 10 μL using SYBR Green PCR Master Mix and 200 nM of each primer. Amplification was carried out in triplicate on a CFX96 Real-Time System (Bio-Rad, Hercules, CA, USA). Primer sequences are provided in Supplementary Table S2. A melt-curve analysis was performed at the end of each run to verify amplification specificity. For the SYBR Green assays, transcript levels were normalized to GAPDH using the ΔCt method and expressed as 2^−ΔCt^. Expression levels in O-αSyn-stimulated sebocytes were subsequently reported as fold change relative to untreated controls, which were assigned a value of 1. Data are presented as mean ± SD from three biological replicates.

### 2.9. Western Blot Analysis

For protein analysis, SZ95 sebocytes were disrupted in RIPA buffer containing a protease/phosphatase inhibitor cocktail and subsequently sonicated. Lysates were centrifuged at 12,000 rpm for 10 min at 4 °C, and the resulting supernatants were stored at −80 °C until use. Protein concentrations were determined spectrophotometrically, after which equivalent amounts of total protein were separated by SDS-PAGE on acrylamide gels and electrotransferred onto nitrocellulose membranes (Bio-Rad Laboratories Srl, Milan, Italy). Successful protein transfer was verified by Ponceau S staining (Sigma-Aldrich). Membranes were rinsed with water and blocked for 10 min at room temperature using EveryBlot Blocking Buffer (Bio-Rad Laboratories Srl, Milan, Italy). They were then incubated overnight at 4 °C with the appropriate primary antibodies, followed by incubation with HRP-conjugated anti-mouse or anti-rabbit IgG secondary antibodies. The complete list of antibodies is provided in Supplementary Table S3. Immunoreactive bands were detected by enhanced chemiluminescence (ECL). GAPDH immunodetection was performed on the same samples as a loading control. Band intensities were quantified by densitometric analysis using the UVITEC Imaging System (Cambridge, UK). Protein expression was normalized to the untreated control, which was assigned a value of 1, and results are reported as mean ± SD from three biological replicates.

### 2.10. Immunofluorescence Analysis

#### 2.10.1. Cell Culture Analysis

SZ95 sebocytes were fixed either in 4% paraformaldehyde followed by permeabilization with 0.1% Triton X-100, or alternatively in cold methanol. After fixation, the cells were incubated with primary antibodies directed against αSyn, PPARγ, BLIMP1, K7, EMA, and PLIN2/ADFP. Immunoreactivity was detected using Alexa Fluor 488- or Alexa Fluor 555-conjugated goat anti-rabbit or goat anti-mouse secondary antibodies (Thermo Fisher Scientific). Coverslips were mounted with ProLong Gold Antifade Mountant containing DAPI (Invitrogen) to stain nuclei. Fluorescence images were acquired using a CCD camera mounted on a Zeiss fluorescence microscope (Zeiss, Oberkochen, Germany). Quantitative assessment of BLIMP1, K7, EMA, and PLIN2 fluorescence intensity was carried out with ZEN 2.6 (Blue Edition; Zeiss, Oberkochen, Germany), and values were reported as mean fluorescence intensity per cell ± SD. For PPARγ, the proportion of cells displaying nuclear immunopositivity was determined by calculating the percentage of positively stained nuclei relative to the total number of cells, with data presented as mean percentage ± SD. Results were obtained from three biological replicates.

#### 2.10.2. Skin Samples Image Analysis

Skin sections were sequentially incubated in: (1) 1% bovine serum albumin (BSA) and 0.1% Triton X-100 for 30 min; (2) a mixture of primary antibodies (2 h at 37 °C and overnight at room temperature), including rabbit anti-αSyn (1:2000, S3062, Merck) with mouse anti-PLIN2 (1:400) or alternatively with mouse anti-PPARγ (1:50). Sections were then washed and incubated for 2 h with two different secondary antibody mixes diluted in 0.1% BSA: (i) goat anti-rabbit Alexa 555 (1:500, Invitrogen) and (ii) goat anti-mouse Alexa Fluor 488 (1:500, Invitrogen). Sections were counterstained for 10 min with DAPI (1:5000, Invitrogen) and mounted using Fluorsave (Merck).

The images were acquired using a Zeiss LSM980 confocal microscope (Zeiss, Oberkochen, Germany). To obtain quantitative data, high resolution confocal images were analyzed using Arivis Pro 4.2 software, applying a “blob finder” pipeline associated with a “particle enhancement” denoising method. Mean intensities and volumes (µm^3^) were quantified for PLIN2 and PPARγ and the intersection volumes between PLIN2, PPARγ, αSyn with nuclei were calculated using an “object math pipeline”, which can automatically quantify the volume of the overlapping conditions between two reconstructed objects and can be applied to any kind of particle.

### 2.11. Nile Red Assay

Following treatment, SZ95 sebocytes were rinsed twice with PBS and incubated with 1 μg/mL Nile red (NR) for 10 min at 37 °C in a humidified 5% CO_2_ atmosphere. Fluorescence was subsequently measured using a DTX 880 Multimode Detector (Beckman Coulter Srl, Milan, Italy). Neutral lipid content was assessed at excitation/emission wavelengths of 485/574 nm, whereas polar lipids were measured at 535/625 nm. Fluorescence values were normalized to the untreated control, which was assigned a value of 1. Data are presented as mean ± SD from three biological replicates. Statistical analyses were performed using GraphPad Prism (GraphPad v11 Software). A two-way ANOVA followed by Tukey’s multiple-comparisons test was applied, with *p* ≤ 0.05 considered statistically significant.

For the microscopic assessment of Nile Red staining, SZ95 sebocytes were fixed in 4% paraformaldehyde and subsequently permeabilized using 0.1% Triton X-100. The cells were then exposed to a 1:1000 dilution of Nile Red for 30 min, rinsed with PBS, and stained with DAPI to visualize nuclei.

### 2.12. Lipid Extraction and Sample Preparation

SZ95 sebocytes collected at 7 days of treatment with 10 µg/mL O-αSyn were suspended in distilled water and frozen and thawed 3 times to crack cell membranes. After centrifugation, the amount of protein present in the cell lysate was determined by Bradford’s assay. The extraction was performed with a methanol/chloroform mixture (2/1 *v*/*v*) in the presence of a mixture of deuterated internal standards. BHT was added to prevent autoxidation. After a centrifugation step, the upper phase was transferred into a vial, and the lipid extract was dried under nitrogen flow and suspended in 200 µL isopropanol prior to analysis. The samples were diluted prior to analysis, as reported in previous studies [34,35].

### 2.13. GCMS

The analysis of lipid extracts using Gas Chromatography–Mass Spectrometry (GCMS) enabled the determination of free fatty acids (FFAs) and cholesterol. Details on the instrumental conditions employed are described in the previous studies [34,35].

### 2.14. LCMS

Reversed Phase-High Performance Liquid Chromatography (RP-HPLC) was used for the chromatographic separation of cholesterol sulfate (CHS), ceramides, glucosylceramides, also known as hexosylceramides (HexCers), long-chain FFAs (27–30 carbon atoms) and fatty acyl esters of hydroxy fatty acids (FAHFA) in negative ESI (-ESI) ion mode, and of cholesterol esters (CEs), triglycerides (TGs) and diglycerides (DGs) in positive ion mode (+ESI). Hydrophilic Interaction Liquid Chromatography (HILIC) was used to separate and detect phospholipids, i.e., phosphatidylcholines (PCs), lysophosphatidylcholines (LPCs), ether-linked phosphatidylcholines (PCs O-), phosphatidylethanolamines (PEs) and sphingomyelins (SMs) in +ESI, and PEs, ether-linked phosphatidyl-ethanolamines (PEs O-), phosphatidylinositols (PIs) and phosphatidylglycerols (PGs) in -ESI mode. The RP-HPLC instrumental conditions employed are described by Maiellaro et al., 2024 [35], while details about HILIC analysis are described by Cavallo et al., 2025 [34]. Sample ionization was performed by the ESI Dual Agilent Jet Stream (AJS) interface connected to the 6545 Quadrupole Time of Flight (Q-TOF) mass spectrometer (Agilent Technologies, Santa Clara, CA, USA), as previously reported [34,36].

### 2.15. Statistical Analysis

Data were presented as mean ± SD of three biological replicates. Statistical significance was assessed using a paired Student’s *t*-test or ANOVA followed by Tukey’s multiple comparison test using GraphPad Prism (GraphPad Software, Boston, MA, USA). For skin biopsies, data were expressed as median ± IQR and analyzed with the Mann–Whitney test. The minimal level of significance was *p* < 0.05.

For lipidomics-derived data, mole amounts were calculated by multiplying the ratio of the analyte peak area to that of the corresponding class-labeled iSTDs by the known quantity of the respective iSTD, as reported in Supplementary Table S1. Lipid abundances in sebocytes were expressed as pmol normalized to cell count.

Statistical analyses were performed using XLSTAT advanced version 2025.2.0 software (Addinsoft, New York, NY, USA) across three biological replicates, each comprising one control and one O-αSyn-treated sample. To identify differential lipid expression between the control and treated SZ95 cells, we applied the Differential Expression module within a paired parametric framework, matching each treated sample directly to its corresponding control. This approach was selected based on the continuous quantitative nature of lipidomic data and the exploratory objective of identifying lipid species modulated by O-αSyn exposure, thereby maintaining high sensitivity to biologically relevant effect sizes. Given the number of lipid species (284) and the small sample size, no post hoc multiple testing correction was applied. From a methodological standpoint, this approach was chosen to prevent an inflation of type II errors (false negatives) in a discovery-driven context, ensuring that subtle but biologically meaningful lipid modulations were not obscured, with the understanding that identified candidates serve as targets for subsequent validation.

Differentially regulated lipids were identified based on the combined criteria of a significance threshold of *p* < 0.05 and a fold-change threshold (FC ≥ 1.25). To simultaneously evaluate the statistical significance and magnitude of these modulations, results were visualized using volcano plots mapping −log10(*p*-value) against log2-transformed FC values.

## 3. Results

### 3.1. O-αSyn Exposure Does Not Affect Viability and Proliferation of SZ95 Cells

**Figure 1:**
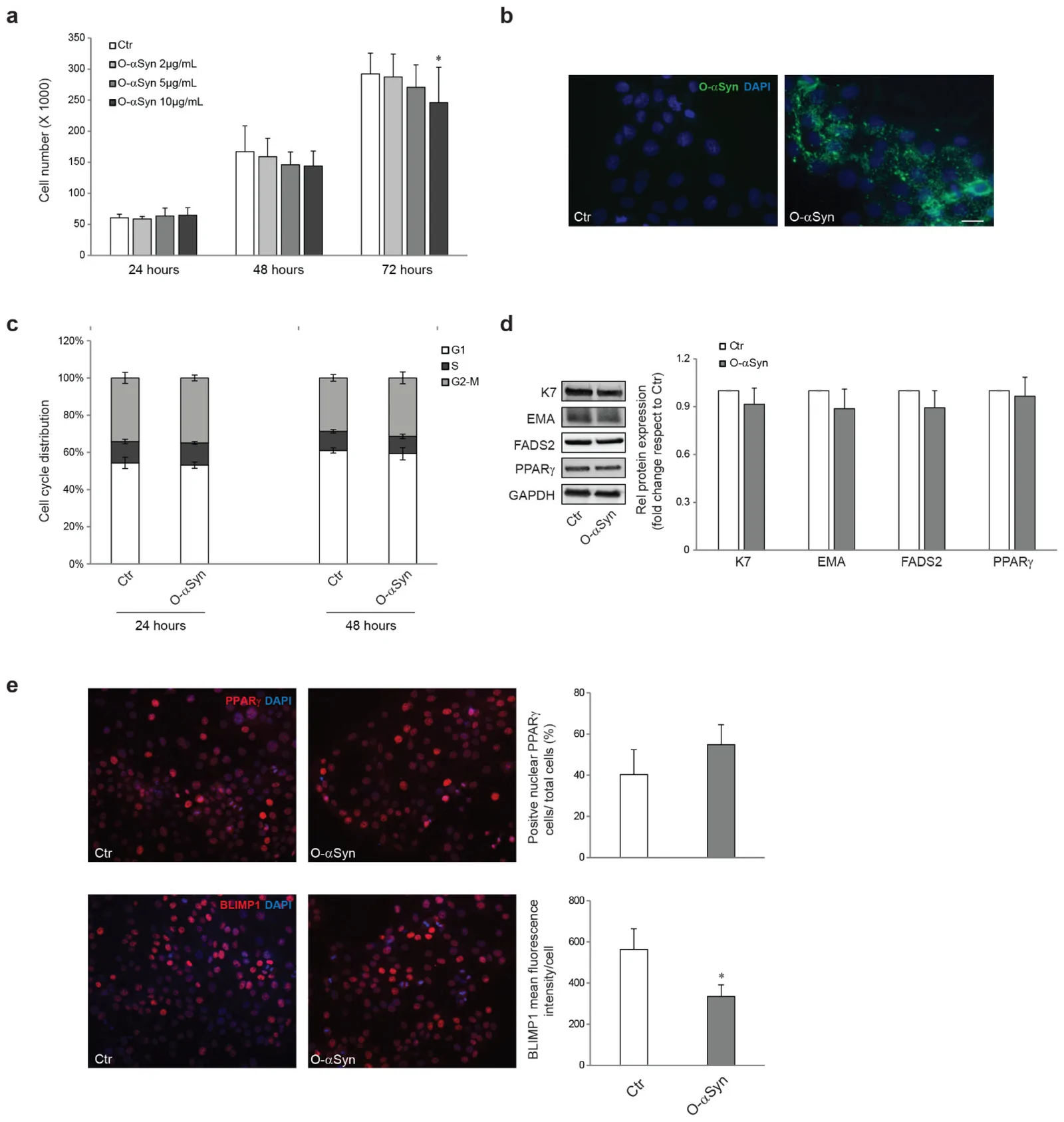
Viability and proliferation of SZ95 sebocytes after O-αSyn exposure and short-term effects on inflammation, lipid metabolism and differentiation. (**a**) Cell counts analysis performed in SZ95 sebocytes treated with O-αSyn (2, 5, and 10 µg/mL) for 24, 48, and 72 h. (**b**) Internalization of O-αSyn evaluated by immunofluorescence analysis on SZ95 cells treated with O-αSyn (10 µg/mL) for 24 h. Nuclei were counterstained with DAPI. Scale bar: 20 µm. (**c**) Cell cycle distribution evaluated by flow cytometric analysis on SZ95 treated with O-αSyn (10 µg/mL) for 24–48 h. The bar graph shows the distribution of cells among the different phases of the cell cycle. (**d**) Western blot analysis of K7, EMA, FADS2, and PPARγ in SZ95 cells treated with O-αSyn (10 µg/mL) for 48 h. Representative blots are shown. GAPDH was used as an endogenous loading control. Densitometric scanning of band intensities was performed to quantify changes in protein expression. Data are expressed as the fold change with respect to untreated control cells (control value taken as 1-fold in each case). (**e**) Immunofluorescence and quantitative analysis of PPARγ and BLIMP1 staining in SZ95 cells treated with O-αSyn (10 µg/mL) for 48 h. At least 450 cells for PPARγ and BLIMP1 were analyzed from three biological replicates. All the data represent the mean ± SD. Statistical significance was determined using Student’s *t*-test. BLIMP1: * *p* = 0.027 vs. untreated control. Nuclei were counterstained with DAPI. Scale bar: 50 µm.

Flow cytometry-based cell counting was performed on SZ95 sebocytes exposed to alpha-synuclein oligomers (O-αSyn) at different concentrations (2, 5, and 10 µg/mL) for 24, 48 or 72 h. Results revealed a slight decrease in sebocyte number only at the highest concentration after 72 h (Figure 1a). Concurrent cell counting experiments using trypan blue staining showed no signs of toxicity at any of the tested concentrations. A wide cytoplasmic distribution of O-αSyn was shown by immunofluorescence analysis (Figure 1b). Considering these results, the 10 µg/mL dose was selected for subsequent experiments. Consistent with the cell counting data, flow cytometry analysis of the cell cycle, performed after 24 and 48 h of O-αSyn treatment, revealed no significant changes in the distribution of the different cell cycle phases (Figure 1c).

### 3.2. Short-Term Effects of O-αSyn on Inflammation, Lipid Metabolism and Differentiation in SZ95 Sebocytes

SZ95 sebocytes exposed to O-αSyn for 24 h did not show significant changes in the expression of genes involved in inflammation, differentiation, and lipid metabolism (Supplementary Table S1). Parallel Western blot analysis performed at 48 h confirmed the invariant expression of sebocyte differentiation markers such as keratin 7 (K7), epithelial membrane antigen (EMA), fatty acid desaturase 2 (FADS2), and peroxisome proliferator-activated receptor gamma (PPARγ). The latter one is a transcription factor that plays a crucial role in sebocyte function, particularly in regulating lipogenesis and sebocyte differentiation [37,38,39,40,41,42,43] (Figure 1d). The results of the immunofluorescence analysis of PPARγ were in line with those of Western blot analysis. However, the analysis revealed a tendency toward increased nuclear staining, which may indicate nuclear translocation and transactivation (Figure 1e). Treatment with O-αSyn caused a noticeable decrease in the transcription factor B-lymphocyte-induced nuclear maturation protein 1 (BLIMP1) staining, which can be a hallmark of promoted sebocyte differentiation (Figure 1e).

### 3.3. Long-Term O-αSyn Treatment Modulates Genes Regulating Sebocyte Differentiation, Inflammation, Cell Metabolism and Lipogenesis

**Figure 2:**
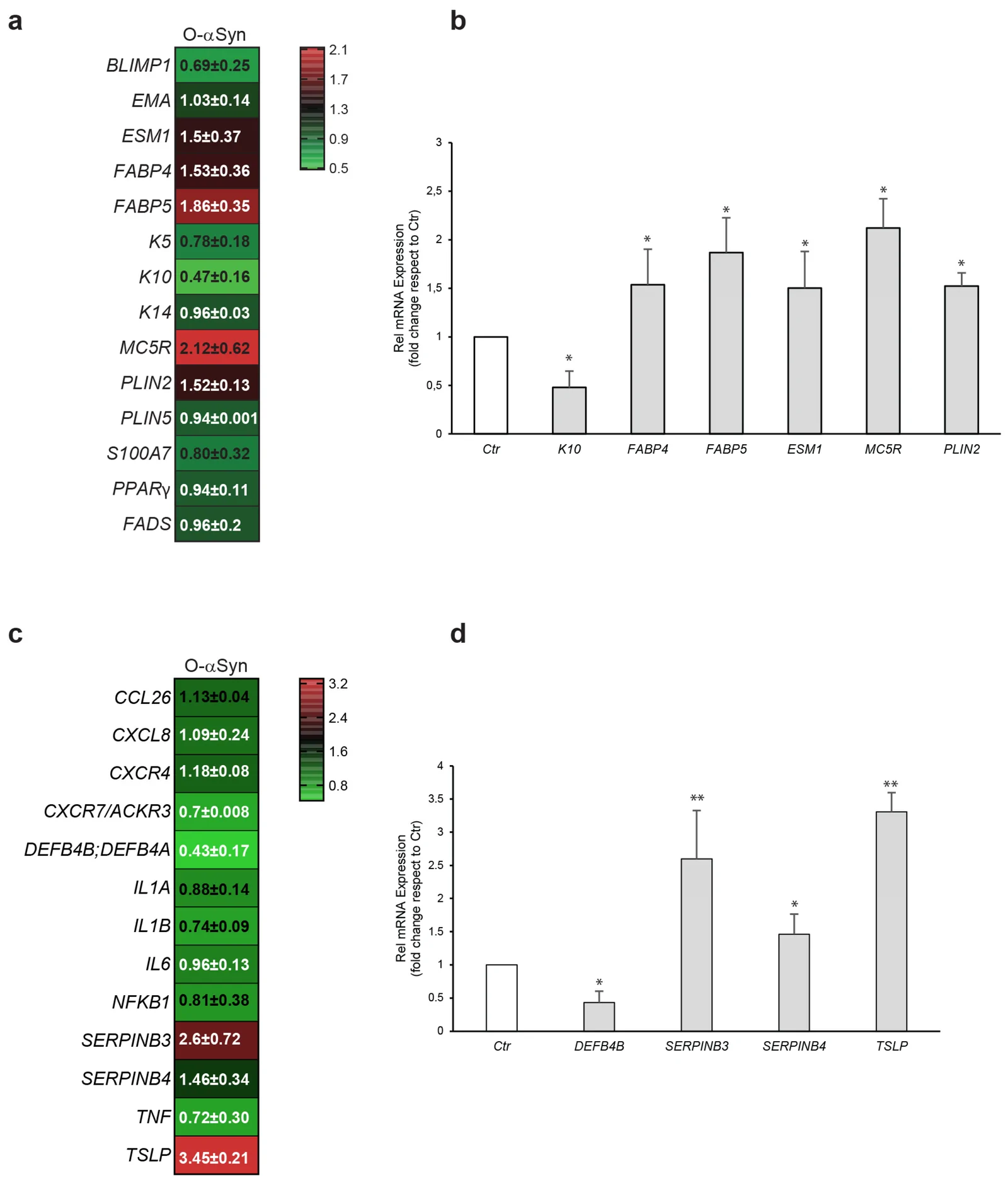
Modulation of genes regulating sebocyte differentiation and inflammation following long-term O-αSyn exposure. (**a**) Array card heatmap illustrating differentially expressed genes involved in sebocyte differentiation in SZ95 sebocytes treated with O-αSyn (10 µg/mL) for 1 week. Red and green shadings represent higher and lower relative log2 fold change expression levels, respectively. (**b**) Quantitative PCR validation of a subset of differentially expressed genes in SZ95 cells treated with O-αSyn (10 µg/mL) for 1 week. (**c**) Array card heatmap illustrating differentially expressed genes involved in inflammatory pathways in SZ95 cells treated with O-αSyn (10 µg/mL) for 1 week. Red and green shadings represent higher and lower relative log2 fold change expression levels, respectively. (**d**) Quantitative PCR validation of a subset of differentially expressed genes in SZ95 cells treated with O-αSyn (10 µg/mL) for 1 week. All mRNA values were normalized against the expression of GAPDH and were expressed relative to untreated control cells. The data in the graphs are mean ± SD of three biological replicates (* *p* < 0.05; ** *p* <0.01 vs. untreated control).

To mimic a chronic condition resembling αSyn accumulation in tissues, SZ95 sebocytes were treated with O-αSyn (10 µg/mL) for one week. The medium was replaced, and treatment was renewed every other day until samples were collected for transcriptomic expression analysis. We first examined the expression of genes involved in differentiation (Figure 2a,b) and inflammation (Figure 2c,d). Heatmap analysis of array card data identified differential expression of multiple markers, subsequently validated by real-time PCR analysis of the most significantly regulated genes. Among differentiation-related genes, K10, FABP4, FABP5, ESM1, MC5R, and PLIN2 showed significant modulation compared to the control (Figure 2b). Notably, late differentiation markers such as MC5R, previously associated with sebum production and sebocyte differentiation [44], were upregulated, whereas the early-stage marker K10 [45] was downregulated. Collectively, these expression changes indicate that O-αSyn stimulation promotes sebocyte differentiation.

In parallel, we examined several inflammatory markers (Figure 2c). The antimicrobial peptide DEFB4B [46] was significantly downregulated, whereas the pro-inflammatory markers SERPINB3, SERPINB4, and TSLP [47] were upregulated (Figure 2d), suggesting a shift toward an inflammatory phenotype of sebocytes following O-αSyn exposure.

**Figure 3:**
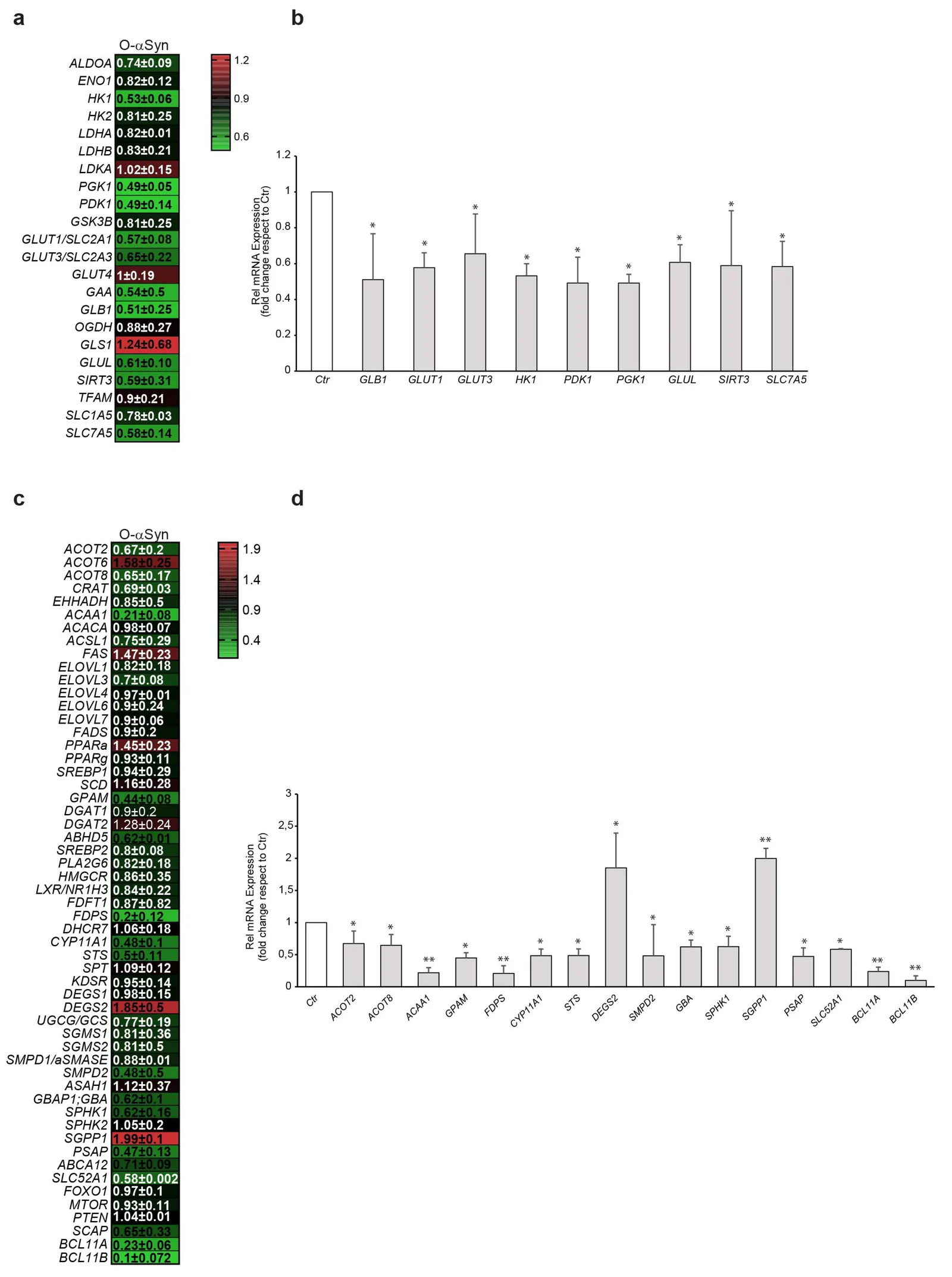
Modulation of genes regulating sebocyte metabolism and lipidogenesis following long-term O-αSyn exposure. (**a**) Array card heatmap illustrating differentially expressed genes involved in energy metabolism in SZ95 sebocytes treated with O-αSyn (10 µg/mL) for 1 week. Red and green shadings represent higher and lower relative log2 fold change expression levels, respectively. (**b**) Quantitative PCR validation of a subset of differentially expressed genes in SZ95 cells treated with O-αSyn (10 µg/mL) for 1 week. (**c**) Array card heatmap illustrating differentially expressed genes involved in lipid metabolism in SZ95 sebocytes treated with O-αSyn (10 µg/mL) for 1 week. Red and green shadings represent higher and lower relative log2 fold change expression levels, respectively. (**d**) Quantitative PCR validation of a subset of differentially expressed genes in SZ95 cells treated with O-αSyn (10 µg/mL) for 1 week. All mRNA values were normalized against the expression of GAPDH and were expressed relative to untreated control cells. The data in the graphs are mean ± SD of three biological replicates (* *p* < 0.05; ** *p* <0.01 vs. untreated control).

Next, we extended the analysis to genes associated with metabolic pathways (Figure 3a,b) and lipid metabolism (Figure 3c,d). Heatmap analysis revealed extensive transcriptional changes in glycolysis- and mitochondrial-related genes (Figure 3a). Real-time PCR validation confirmed a significant downregulation of several transcripts, such as GLB1, GLUT1, GLUT3, HK1, PDK1, PGK1, GLUL, SLC7A5, and SIRT3 (Figure 3b). Similarly, genes involved in lipid synthesis and remodeling, such as ACAA1, GPAM, BCL11A/B, and several regulators of sphingolipid metabolism (e.g., GBA, SGPP1, and DEGS2), exhibited significant expression changes (Figure 3c). Quantitative PCR confirmed a significant downregulation of most of these genes, whereas a subset, including the sphingosine phosphatase SGPP1, and the sphingolipid desaturase DEGS2, was upregulated (Figure 3d).

Taken together, these data indicate that sebocytes exposed to O-αSyn undergo profound shifts in metabolic and lipid-related gene expression.

### 3.4. Long-Term Treatment with O-αSyn Alters the Expression of SZ95 Sebocyte Differentiation Markers

**Figure 4:**
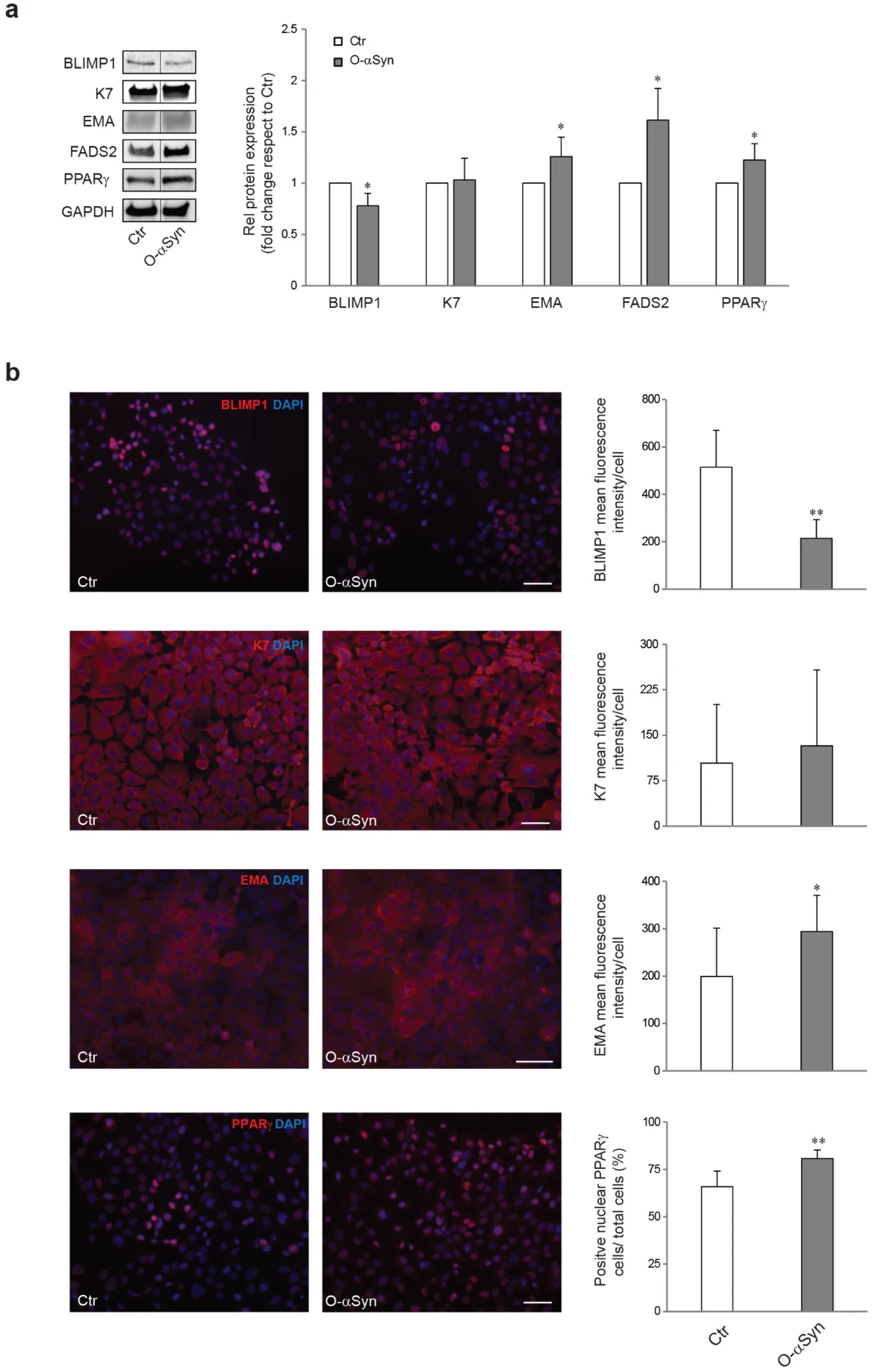
Alteration of the expression of SZ95 sebocyte differentiation markers following long-term O-αSyn exposure. (**a**) Western blot analysis of BLIMP1, K7, EMA, FADS2, and PPARγ in SZ95 sebocytes treated with O-αSyn (10 µg/mL) for 1 week. Representative blots are shown. GAPDH was used as an endogenous loading control. Densitometric scanning of band intensities was performed to quantify changes in protein expression. Data represent the mean ± SD of three biological replicates and are expressed as the fold change with respect to untreated control cells (control value taken as 1-fold in each case) (* *p* < 0.05 vs. untreated control). (**b**) Immunofluorescence and quantitative analysis of BLIMP1, K7, EMA and PPARγ signals in SZ95 sebocytes treated with O-αSyn (10 µg/mL) for 1 week. At least 600, 700, 1000, 800 cells were evaluated for BLIMP1, K7, EMA and PPARγ, respectively. Data are presented as the mean ± SD and were obtained from three biological replicates. Statistical significance was determined using Student’s *t*-test. BLIMP1: ** *p* = 0.004; EMA: * *p* = 0.044; PPARγ: ** *p* = 0.004 vs. untreated control. Nuclei were counterstained with DAPI. Scale bar: 50 µm.

Sebaceous differentiation is characterized by a specific pattern of protein expression at different stages of the process [45,48]. The expression of the early differentiation marker K7 was unaffected after exposure to O-αSyn. Conversely, the treatment caused a reduction in BLIMP1. The specific deletion of BLIMP1 has been linked to differentiation defects in multiple epidermal compartments, including the SG [45,49,50,51]. O-αSyn treatment also resulted in a significant induction of EMA and FADS2, which are both involved in the middle-to-late stages of sebocyte differentiation [45,52,53,54]. Similarly, PPARγ, which promotes the differentiation of early sebocytes into mature ones, was induced (Figure 4a). Immunofluorescence and quantitative analysis confirmed the reduction in BLIMP1, the invariance of K7, and the increase in EMA signals following O-αSyn exposure. Furthermore, the nuclear translocation of PPARγ was significantly induced, suggesting promotion of its transactivation (Figure 4b).

### 3.5. Long-Term Treatment with O-αSyn Induces the Expression of Proteins Involved in Lipid Metabolism

**Figure 5:**
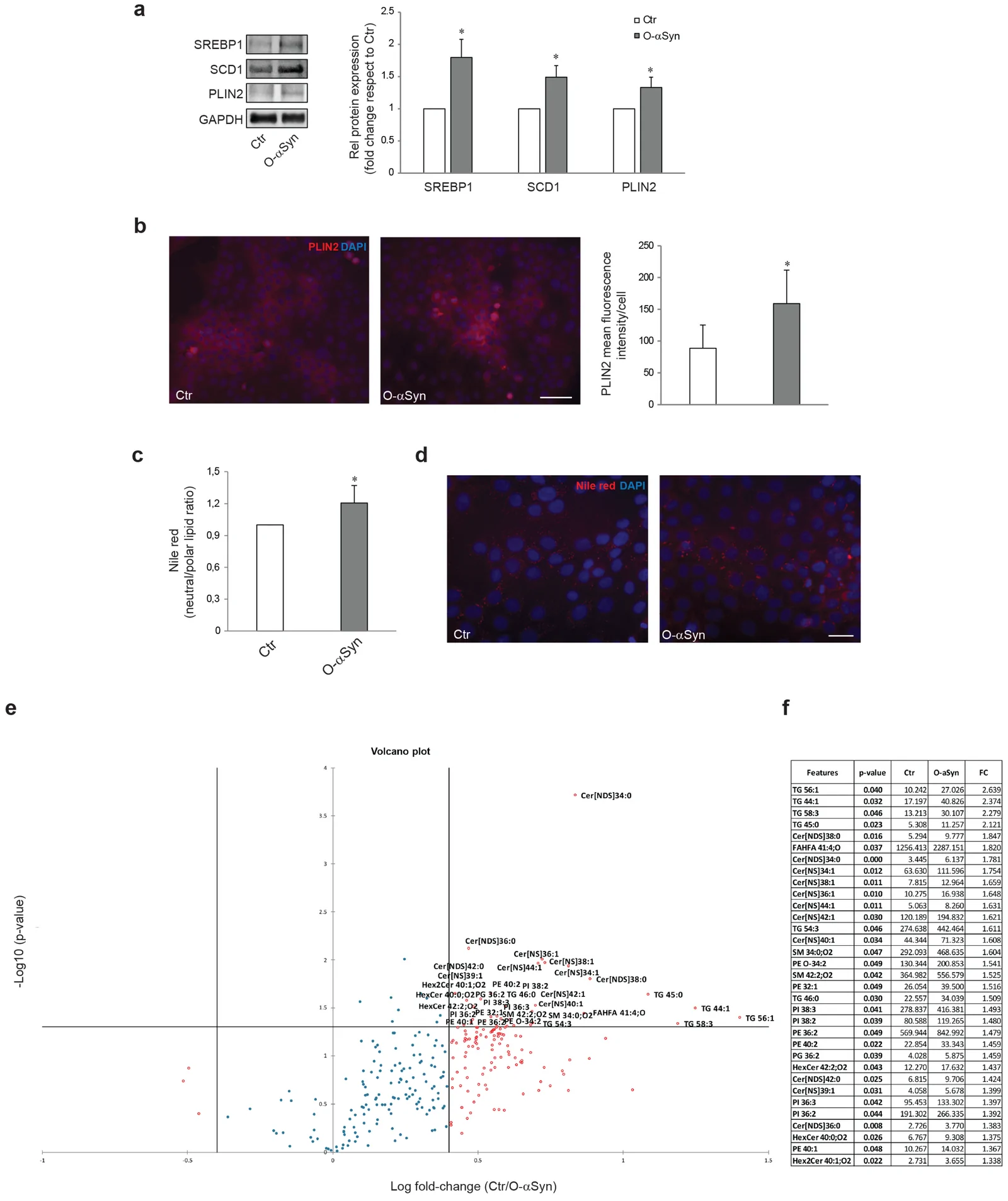
Long-term O-αSyn exposure induces the expression of proteins involved in lipid metabolism and storage alters lipid composition. (**a**) Western blot analysis of SREBP1, SCD1, and PLIN2 in SZ95 sebocytes treated with O-αSyn (10 µg/mL) for 1 week. Representative blots are shown. GAPDH was used as an endogenous loading control. Densitometric scanning of band intensities was performed to quantify changes in protein expression. Data are expressed as the fold change with respect to untreated control cells (control value taken as 1-fold in each case) (* *p* < 0.05 vs. untreated control). (**b**) Immunofluorescence and quantitative analysis of PLIN2 in SZ95 sebocytes treated with O-αSyn (10 µg/mL) for 1 week. At least 1000 cells were evaluated from three biological replicates. Data are presented as the mean ± SD. Statistical significance was determined using Student’s *t* test. * *p* = 0.049 vs. untreated control. Nuclei were counterstained with DAPI. Scale bar: 50 µm. (**c**) Determination of neutral lipids, polar lipids and neutral-to-polar lipid ratio by Nile red assay in SZ95 sebocytes treated with O-αSyn (10 µg/mL) for 1 week. The data represent the mean ± SD of three biological replicates (* *p* < 0.05 vs. untreated control). (**d**) Microscopical analysis of Nile red staining performed on SZ95 sebocytes treated with O-αSyn (10 µg/mL) for 1 week. Nuclei were counterstained with DAPI. Scale bar: 20 µm. (**e**) Volcano plot showing differential lipid expression between control and O-αSyn-treated SZ95 sebocytes. The x-axis represents the log2 FC (Ctr/O-αSyn), while the y-axis represents –log10(*p*-value). Each dot corresponds to a single lipid species. Lipids significantly modulated by O-αSyn treatment are colored in red and labeled, based on a combined threshold of *p* < 0.05 and FC ≥1.25. (**f**) Summary Table of the 33 lipid species reaching statistical significance, with both *p*-values <0.005 and fold changes (FC) >1.32.

We then evaluated the impact of O-αSyn treatment on the expression of key proteins involved in lipogenesis. O-αSyn induced the expression of sterol response element-binding protein-1 (SREBP1), a transcription factor that plays a crucial role in the synthesis of key sebum lipids, including TGs, FFAs and cholesterol [55,56]. Additionally, O-αSyn exposure increased the levels of stearoyl-CoA desaturase 1 (SCD1), an enzyme catalyzing the conversion of saturated fatty acids (SFAs) into monounsaturated fatty acids (MUFAs) in concert with FADS2 [52,57]. O-αSyn also increased the expression of perilipin 2 (PLIN2), a key regulator of lipid droplet (LD) formation that influences lipid organization and storage within sebaceous cells—processes essential for proper sebocyte differentiation and overall sebum production [58] (Figure 5a).

Immunofluorescence and quantitative image analysis of PLIN2 confirmed the higher expression of PLIN2 in treated cells in comparison with control sebocytes (Figure 5b). The Nile red assay also enabled both visualization and quantification of neutral lipids (e.g., TGs and sterol esters in LDs) and polar lipids (e.g., phospholipids in membranes) stored within sebocytes [59]. Notably, treatment with O-αSyn significantly increased the neutral-to-polar lipid ratio, suggesting a higher deposition of neutral storage lipids in LDs (Figure 5c). Microscopical analysis of Nile red staining revealed the presence of larger dots in sebocytes treated with O-αSyn compared to control cells, further supporting increased lipid accumulation (Figure 5d).

### 3.6. Long-Term Effects of O-αSyn Treatment on Lipid Composition and Metabolism

To investigate the long-term impact of O-αSyn on sebocyte lipid metabolism, lipid extracts were analyzed using a combined GCMS and LCMS approach, allowing the identification and quantification of 284 lipid species (Supplementary Table S4). Statistical analyses revealed that O-αSyn treatment significantly modulated distinct lipidomic metabolic subsets. As shown in Figure 5e, O-αSyn-treated SZ95 sebocytes displayed a clear trend toward increased abundance of TGs, ceramides, HexCers, and selected phospholipids, including SMs, PEs, and PIs, compared with controls. Specifically, the differential lipidomic analysis identified 33 lipid species significantly modulated by O-αSyn treatment in SZ95 sebocytes based on combined statistical and FC criteria (Figure 5f). Significance was confirmed using an empirical Bayes framework, with false discovery rates (FDRs) controlled using the Benjamini–Hochberg procedure. Most of the significantly altered lipids showed increased abundance in O-αSyn-treated cells compared with controls, with FC ranging from 1.25 to 2.64. The most pronounced increases were observed for TGs, i.e., TG 56:1, TG 44:1, TG 58:3, and TG 45:0, which displayed FCs above 2.0. In addition to these neutral lipids, several ceramide species were significantly upregulated, encompassing both dihydrosphingosine-based (Cer[NDS]) and sphingosine-based (Cer[NS]) ceramides, with FCs between 1.4 and 1.8. Significant changes were also detected in complex sphingolipids, such as HexCers and SMs, as well as in selected phospholipids. These species displayed moderate but consistent increase in abundance (FC between 1.3 and 1.6).

**Figure 6:**
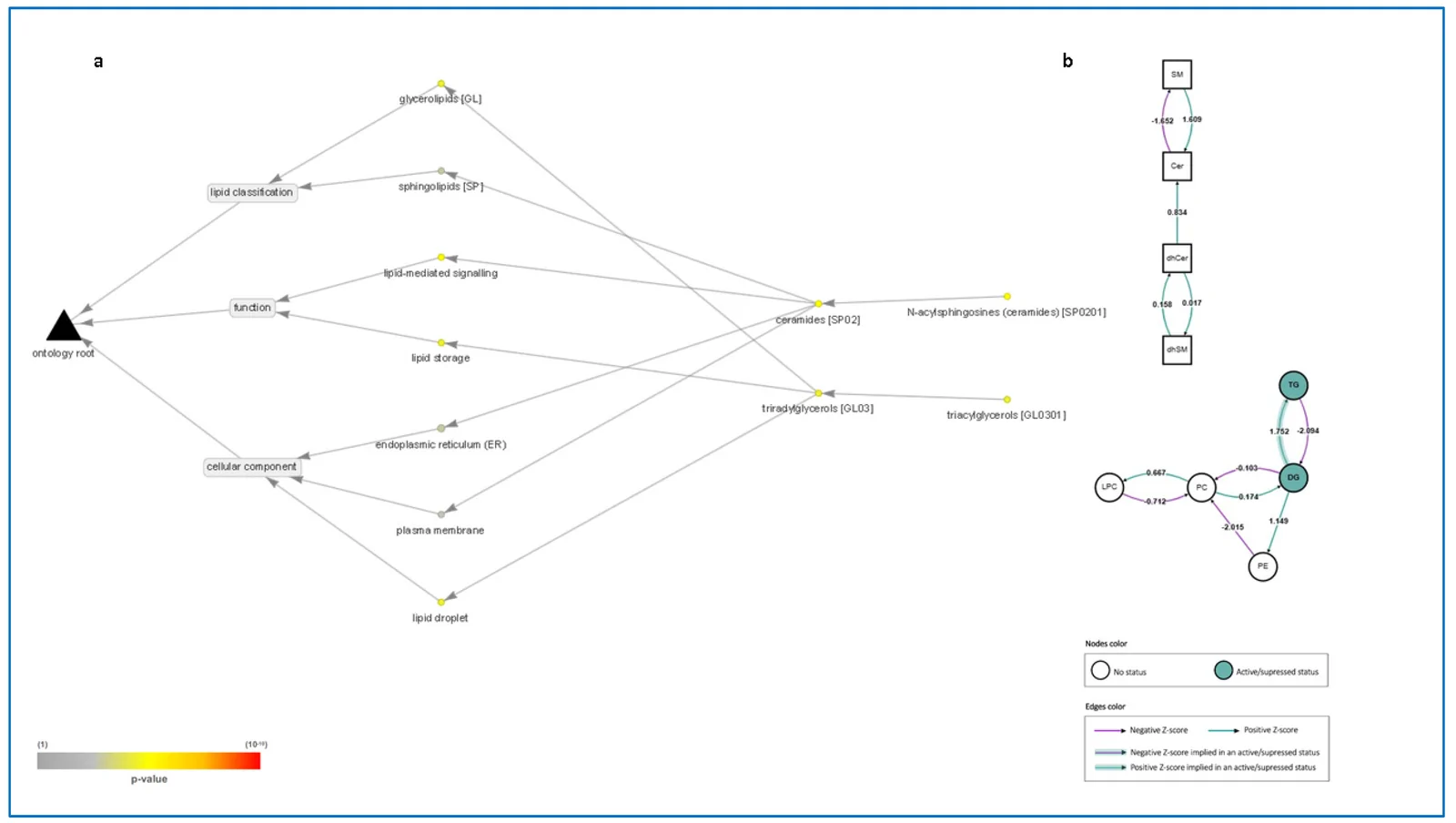
Lipidomic ontology enrichment and pathway analysis. (**a**) LION/web enrichment analysis of lipids in O-αSyn-treated SZ95. The color scale represents statistical significance. (**b**) Activated lipid pathways suggested by BioPan in SZ95 sebocytes. Purple lines indicate a negative Z-score, while blue lines indicate a positive Z-score. Blue overlays represent significant changes in the activated or suppressed status of these pathways.

Furthermore, lipidomic data were analyzed using the web-based Lipid Ontology enrichment tool [60,61] which identified significantly enriched LION terms (ranking mode) in SZ95 cells (Figure 6a).

The analysis revealed significant enrichment of TGs and N-acylsphingosines (ceramides), consistent with the relative quantification observed at the lipid class level (Figure 5e,f). Glycerolipids, specifically TGs, were associated with “lipid storage” functions and localization to “lipid droplets”. Additionally, sphingolipids, particularly ceramides, were associated with “lipid-mediated signaling” functions and localization to the “endoplasmic reticulum (ER)” and “plasma membrane”. The lipidomic pathway analysis, performed via BioPAN [62] (Figure 6b), revealed significant alterations in lipid metabolism in O-αSyn-treated SZ95 sebocytes. Specifically, glycerophospholipid metabolism was predicted to shift toward TG synthesis from DGs. This metabolic flux may be associated with our observation of increased SCD1 and PLIN2 expression, together with the accumulation of neutral lipids detected by Nile Red staining (Figure 5a–d). Within the sphingolipid pathway, BioPAN predicted increased synthesis of dihydroceramide (dhCer). This result is compatible with our gene expression data (Figure 3d), which show a significant upregulation of *DEGS2*, the key enzyme responsible for the metabolic conversion of dhCer to ceramide. Furthermore, while the gene expression analysis revealed only mild modulations in *SGMS1* and *SGMS2* (Figure 3c), these subtle changes may contribute to the observed modulations of SM and ceramide levels.

Overall, these findings indicate that O-αSyn promotes the accumulation of specific TGs and ceramides and alters membrane lipid composition, thereby reflecting an extensive perturbation of sebocyte lipid homeostasis.

### 3.7. Preliminary Study on the Connection Between αSyn and PLIN2/PPARγ in the SGs of Skin Biopsies from PD Patients

**Figure 7:**
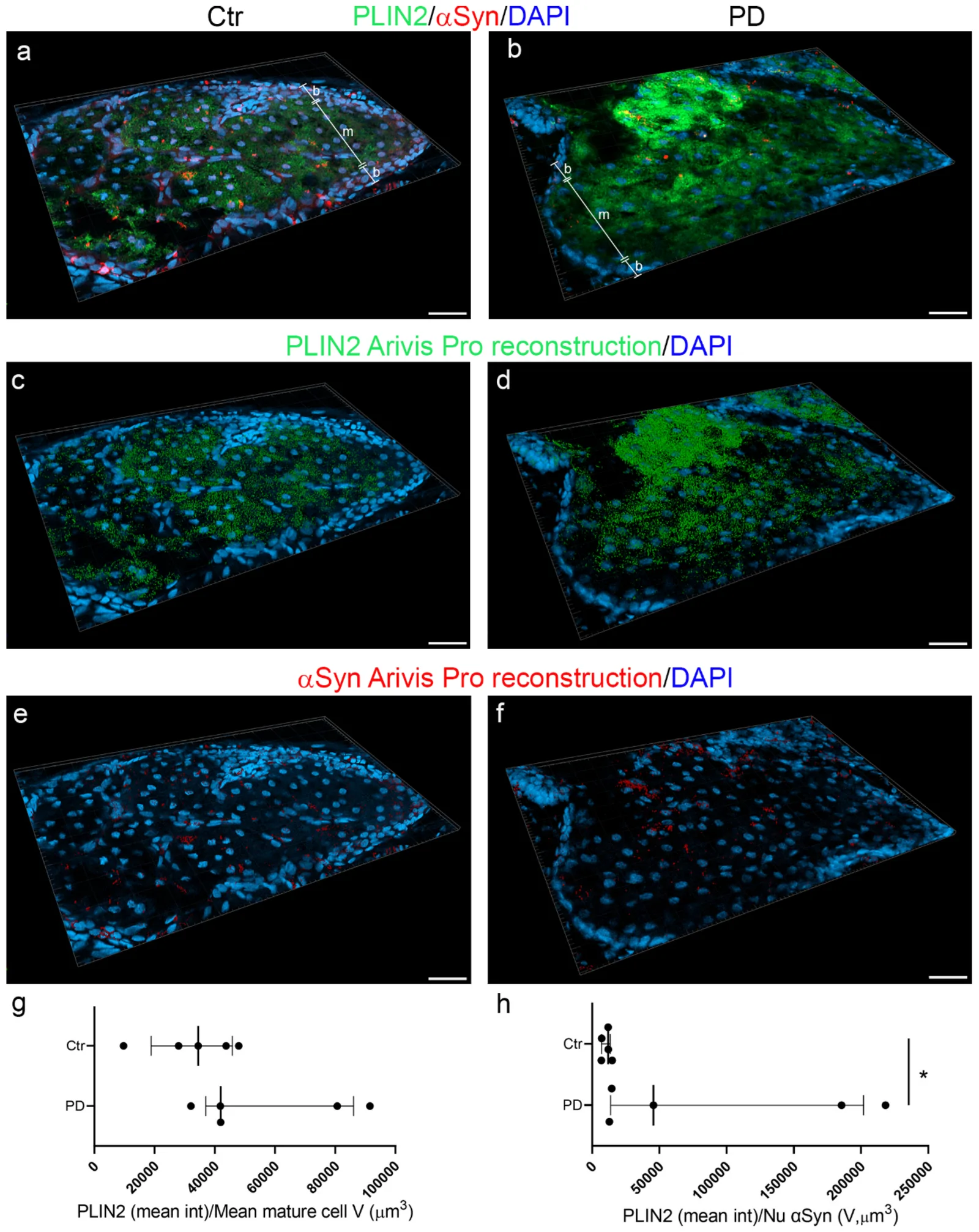
αSyn and PLIN2 confocal and Arivis Pro software analysis in sebaceous glands obtained from control and PD patients. (**a**,**c**) In controls, PLIN2 is mainly distributed in the cytoplasm of mature sebocytes, where lipid droplets are densely packed. (**b**,**d**) In PD, PLIN2 expression is increased in the cytoplasm of mature sebocytes. (**e**,**f**) αSyn staining is also detectable both in the cytoplasm and in the nuclei. (**g**) Quantitative analysis of PLIN2 mean intensity rationalized to the mature sebocytes nuclei volume. (**h**) Graph representing PLIN2 mean intensity rationalized to the αSyn volume inside the nuclei. Data are represented as median ± IQR. * *p* < 0.05. Scale bar: 40 µm. b = basal cells, m =mature cells, Nu = nuclear.

To provide preliminary validation of our in vitro findings, we investigated the relationship between αSyn, PLIN2, and PPARγ in sebaceous glands by comparing skin biopsies obtained from a small cohort (*n* = 5) of patients with PD and age-matched controls (*n* = 5). PLIN2 displayed a compartment-specific distribution, mainly localized in the cytoplasm of mature sebocytes, where LDs are densely packed (Figure 7a–d). Analysis using Arivis Pro 4.2 software revealed a mild increase in cytoplasmic PLIN2 in mature sebocytes of PD patients compared to controls (Figure 7g). In these cells, αSyn staining was also detectable both in the cytoplasm and the nuclei of controls and PD samples (Figure 7e,f). Furthermore, we examined the relationship between PLIN2 and αSyn and observed a significant increase in the ratio of PLIN2 mean intensity relative to the volume of αSyn localized inside the nuclei in PD patients compared to controls (Figure 7h). These preliminary findings suggest a potential link between LD-associated proteins and αSyn in the SGs of patients with PD.

**Figure 8:**
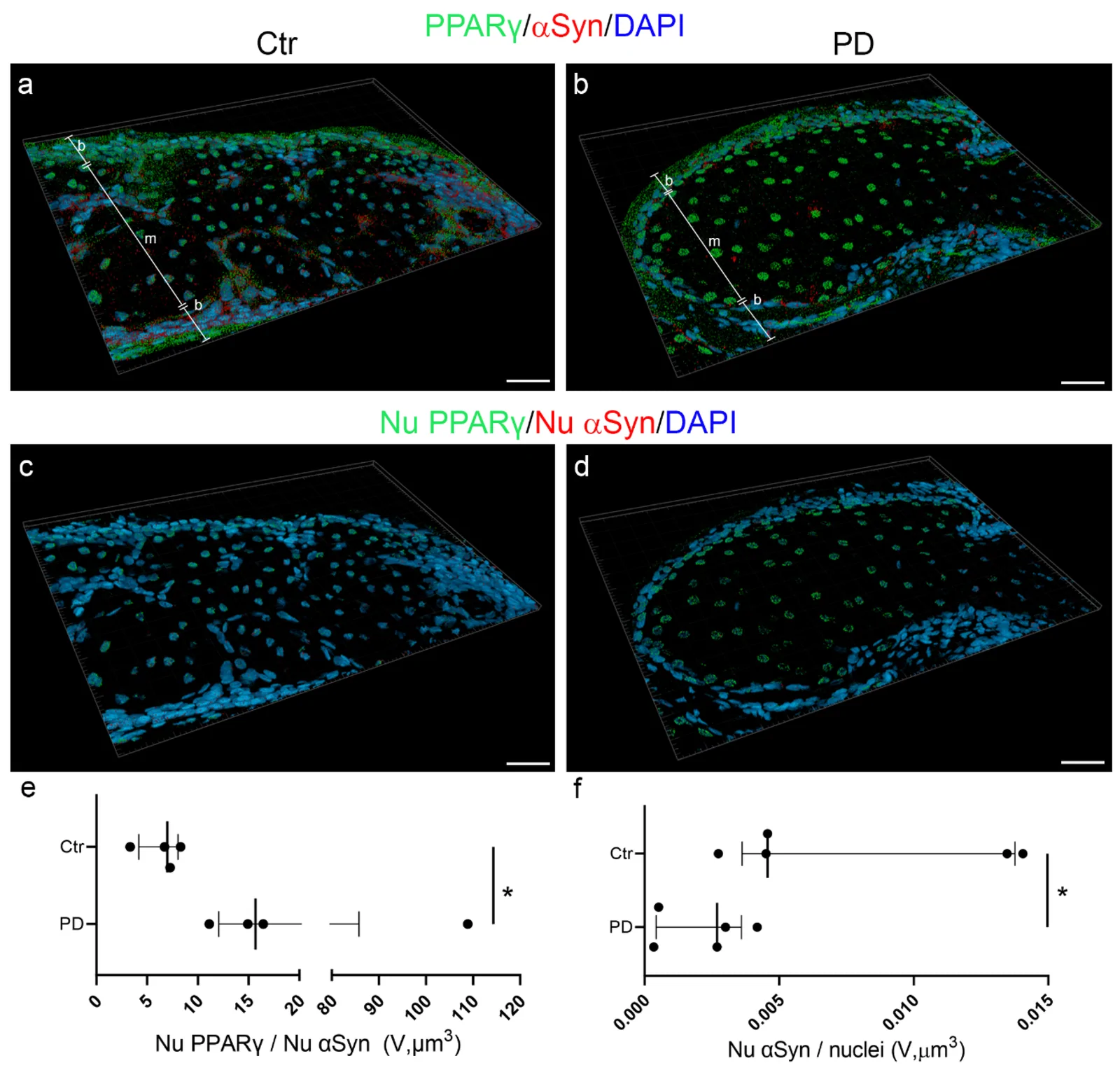
αSyn and PPARγ Arivis Pro software analysis in sebaceous glands obtained from control and PD patients. (**a**,**c**) In control subjects, PPARγ is distributed both in the cytoplasm and in the nucleus of basal sebocytes, whereas in mature sebocytes it is mainly localized in the nucleus. (**b**,**d**) In PD samples, PPARγ expression is increased, predominantly in the nuclei of mature sebocytes, where αSyn is also present. (**e**) Quantitative analysis of nuclear PPARγ rationalized to the nuclear αSyn volume. (**f**) Graph representing the volume of nuclear αSyn rationalized to the volume of nuclei. Data are represented as median ± IQR. * *p* < 0.05. Scale bar: 40 µm. b = basal cells, m = mature cells, Nu = nuclear.

We also explored the differential expression of PPARγ in control and PD subjects. We observed that PPARγ is present in both the cytoplasm and nuclei of basal cells and becomes mainly nuclear in mature sebocytes (Figure 8a,b). Quantitative analyses revealed an increase in nuclear PPARγ relative to nuclear αSyn volume in PD patients compared to controls (Figure 8c–e). More importantly, we also reported a decrease in nuclear αSyn, measured as nuclear αSyn per nuclei, in PD patients (Figure 8c–f).

## 4. Discussion

Among the multiple comorbidities associated with PD, SD affects approximately 50–60% of patients and is considered a premotor feature of the disease [23,63]. However, the mechanisms underlying PD-associated hyperseborrhea remain poorly understood. The accumulation of αSyn in the skin, particularly within the pilosebaceous unit, may interfere with SG function, potentially leading to alterations in both sebum secretion and composition [29]. To our knowledge, this is the first study elucidating the functional alterations in sebocytes resulting from intracellular accumulation of αSyn, with consequent effects on sebum abundance and composition.

Our results indicate that long-term exposure to O-αSyn is associated with increased expression of markers characteristic of mid- to late-stage sebocyte differentiation, suggesting a shift toward a more mature phenotype with enhanced lipid production and accumulation in lipid droplets (LDs) [64,65,66,67,68]. Gene expression analysis revealed upregulation of late differentiation markers together with reduced K10 expression, a marker associated with sebocyte turnover and loss of differentiation [69]. At the protein level, decreased BLIMP1 expression, together with increased EMA, FADS2, and PPARγ, suggests that prolonged O-αSyn exposure may dysregulate sebocyte differentiation. While increased expression of EMA, FADS2, and PPARγ is consistent with enhanced sebocyte maturation and lipogenesis, reduced BLIMP1 may reflect impaired sebaceous gland homeostasis. Indeed, previous studies showed that the loss of BLIMP1 results in glandular abnormalities rather than simply inhibiting sebocyte differentiation [49,51]. The increase in PPARγ expression and its nuclear translocation further support enhanced transcriptional activation of lipogenic pathways. As the master regulator of sebocyte differentiation and lipid synthesis, PPARγ plays a central role in sebaceous gland development and function [38,41,42,70,71,72,73]. In parallel, O-αSyn significantly increased MC5R expression, a receptor involved in sebocyte differentiation and sebaceous lipid synthesis through α-MSH signaling [44,74,75,76,77]. Together, these findings support the hypothesis that αSyn contributes to sebocyte differentiation toward a lipid-producing phenotype.

The inflammatory signature observed following O-αSyn exposure is compatible with a pathological role for αSyn in sebocytes. Increased expression of serpins and TSLP suggests activation of pro-inflammatory pathways, whereas reduced DEFB4B expression may indicate alterations in antimicrobial defense [78]. These findings may be relevant to the altered skin microenvironment described in PD, although the potential relationship between αSyn-induced changes in sebocytes and cutaneous microbial dysbiosis remains to be established. In particular, whether such changes may influence the abundance or composition of *Malassezia*, which has been associated with SD, remains speculative and requires further investigation [12,16,79].

Our findings also suggest that O-αSyn may remodel metabolism in sebocytes. The coordinated downregulation of glycolytic and mitochondrial genes may reflect reduced oxidative metabolism associated with increased anabolic lipid synthesis during sebocyte maturation and lipid storage in LD, similarly to metabolic patterns described in adipocyte differentiation models [80,81,82]. Consistently, reduced expression of ACOT2 and ACOT8, enzymes involved in mitochondrial and peroxisomal β-oxidation [83,84] further supports a reduced oxidative lipid metabolism, although alternative interpretations cannot be excluded. Consistent with this metabolic remodeling, O-αSyn affected several genes involved in lipid metabolism. For example, *GBA* expression was significantly reduced. Interestingly, evidence identifies GBA deficiency as one of the strongest genetic risk factors for PD and links sphingolipid metabolism to α-synucleinopathies [85,86]. Increased *DEGS2* and *SGPP1* were accompanied by altered sphingolipid levels, supporting a broader perturbation of sphingolipid metabolism. Given the central role of sphingolipids, particularly ceramides, in membrane organization, inflammatory signaling, and barrier integrity [87,88,89], these alterations may contribute to sebocyte dysfunction. Alterations in ceramide metabolism have been widely described in PD, although their biological impact appears to depend on the tissue examined and on the specific ceramide species involved [90,91,92]. Indeed, ceramides differing in fatty acyl chain length exert distinct biological functions, and short-chain species such as C16-ceramide have been associated with mitochondrial dysfunction [92]. Therefore, the altered sphingolipid profile observed in our model suggests a metabolic remodeling associated with O-αSyn exposure and could potentially amplify mitochondrial dysfunction and inflammatory responses.

As sebocytes mature, they undergo a progressive accumulation of neutral lipids within LDs, which are essential for sebum build-up and secretion. During this process, PLIN2 promotes both LD biogenesis and stability, thereby regulating intracellular lipid storage [58,93,94,95,96,97]. Increased PLIN2 expression is widely recognized as a marker of sebocyte differentiation toward a lipid-producing phenotype [58]. Our finding that O-αSyn increases the expression of PLIN2 together with the lipogenic regulators SREBP1, SCD1 and FADS2 suggests that O-αSyn may contribute to the promotion of lipid synthesis and storage within LDs. SREBP1 is a master regulator of lipogenesis, whereas SCD1 and FADS2 catalyze the synthesis of mono- and polyunsaturated FFAs, which represent major constituents of sebum [52,55,57]. Consistent with these findings, lipidomic analysis revealed a marked increase in TGs, particularly long-chain and unsaturated species, together with phospholipids, including PEs and PIs. Lipid enrichment and pathway analyses further supported a shift toward a neutral lipid-rich profile, characteristic of differentiating sebocytes. Such lipid remodeling is consistent with the established role of PPARγ in regulating the expression of lipogenesis-associated genes, thereby promoting lipid accumulation and sebocyte differentiation [38,98]. Indeed, TGs, the major lipid component of human sebum, progressively accumulate within LDs as sebocytes mature.

Growing evidence indicates that αSyn perturbs cellular lipid homeostasis through its interaction with phospholipid membranes and LDs [99,100,101,102]. The association of αSyn with PLIN2-coated LDs suggests a bidirectional relationship in which αSyn may influence LD biogenesis and lipid storage, while LDs may buffer αSyn by sequestering soluble protein and modulating its aggregation [100,103]. In PD, alterations in αSyn expression, conformation, or aggregation propensity are frequently associated with disrupted lipid metabolism [104,105,106]. Within this αSyn-PLIN2-LD interplay, the increased PLIN2 expression induced by O-αSyn may favor LD expansion while simultaneously influencing intracellular αSyn distribution and activity. Although this mechanism remains hypothetical, it may provide a plausible explanation for the coordinated changes in sebocyte differentiation, lipid remodeling, and sebum-related lipid composition observed in SZ95 cells.

In addition, we observed reduced nuclear αSyn immunoreactivity in sebaceous glands from PD patients. Emerging evidence suggests a physiological role for nuclear αSyn in the repair of DNA double-strand breaks, although its precise nuclear function remains incompletely understood. Notably, it has been hypothesized that cytoplasmic aggregation of αSyn may decrease its soluble nuclear pool, potentially leading to a loss-of-function mechanism that may promote DNA damage [107,108]. However, these mechanisms remain hypothetical and require further validation. Given the preliminary nature of this skin biopsy study, further investigations in larger and more diverse patient populations, including subjects with SD, are needed to confirm and extend the present findings.

**Figure 9:**
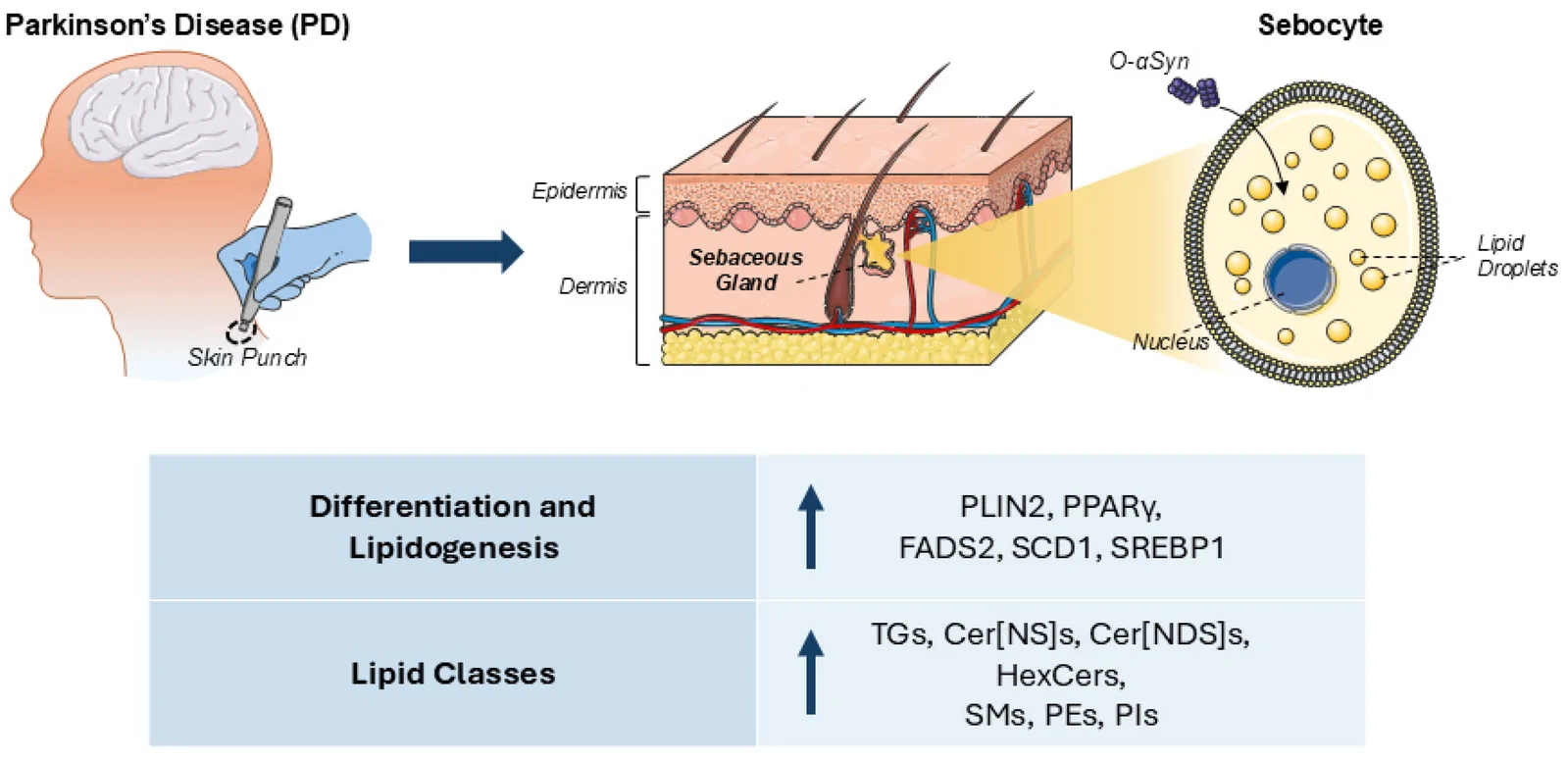
Schematic overview of the effects of intracellular O-αSyn on the SZ95 sebocytes and skin biopsies from PD patients. αSyn exposure is associated with features of sebocyte maturation and lipid remodeling, together with alterations in sebum lipid production and composition.

In conclusion, our study suggests that the exposure to O-αSyn is associated with molecular changes consistent with altered sebocyte differentiation and lipid remodeling. The link between αSyn and lipid dysregulation, suggests that sebocytes may represent unrecognized peripheral targets of αSyn (Figure 9).

## 5. Limitations

This study has some limitations that should be acknowledged. Although the immortalized human sebocyte cell line (SZ95) closely reproduces the morphological, phenotypic, and functional characteristics of sebocytes, further studies using primary human sebocytes and/or organotypic sebaceous gland models will be required to validate and extend our findings.

A further limitation of this study is that the concentration of O-αSyn used was selected based on previous in vitro studies performed in other cell types. Therefore, it may not accurately reflect the actual concentration of oligomeric α-synuclein present in the SG of patients with PD, which remains currently unknown. Consequently, the experimental concentration used may not fully reproduce the in vivo condition.

Finally, our in vivo observations are limited by the relatively small size of the patient cohort. Larger, independent cohorts will be necessary to validate these preliminary findings and to determine their biological, diagnostic, and clinical relevance.

## Figures and Tables

**Table 1:** Demographic and clinical characteristics of the skin biopsy cohort. Individual data are shown for the five patients with Parkinson’s disease (PD) and the five healthy controls (CTRL) who underwent skin biopsy. Motor severity was assessed with the MDS-UPDRS part III in the ON and OFF medication states and staged according to Hoehn and Yahr; disease duration (DD) is reported in years (y) from diagnosis. LEDD was calculated according to Jost et al. [32]. Motor phenotype was classified as tremor-dominant or akinetic-rigid according to Stebbins et al. [33]. Autonomic symptom burden was assessed with the COMPASS-31 questionnaire, and both the total weighted score (range 0–100) and the six weighted domain sub-scores are reported: d1, orthostatic intolerance (0–40); d2, vasomotor (0–5); d3, secretomotor (0–15); d4, gastrointestinal (0–25); d5, bladder (0–10); d6, pupillomotor (0–5). Higher scores indicate greater autonomic symptom burden. Seborrheic dermatitis (SD) and dermatological comorbidities were recorded from clinical history and dermatological examination. Clinical and autonomic measures were not applicable to controls. Abbreviations: CTRL, healthy control; d, domain; H&Y, Hoehn and Yahr stage; LEDD, levodopa equivalent daily dose (mg/day); M, male; MDS-UPDRS, Movement Disorder Society-sponsored revision of the Unified Parkinson’s Disease Rating Scale; NA, not available; PD, Parkinson’s disease; y, years.

Subject	Age (y)	Sex	DD (y)	UPDRS ON/OFF	H&Y	LEDD	Disease Phenotype	COMPASS-31 Total Score	d1	d2	d3	d4	d5	d6	SD
PD 1	60	M	4	21/38	2	1075	Akinetic rigid	25.92	12	0	0	10.7	2.22	0.99	No
PD2	45	M	5	24/58	2	1625	Tremor dominant	20.44	16	0	0	2.67	1.11	0.66	No
PD 3	60	M	2	13/23	1	588	Tremor dominant	1.55	0	0	0	0.89	0	0.66	No
PD 4	47	M	1	19/NA	1	225	Tremor dominant	9.37	0	0	2.14	4.46	1.11	1.66	No
PD 5	58	M	5	21/NA	2	100	Akinetic rigid	16.74	0	0	4.28	5.36	4.44	2.66	No
CTRL 1	66	M	-	-	-	-	-	NA	NA	NA	NA	NA	NA	NA	No
CTRL 2	60	M	-	-	-	-	-	NA	NA	NA	NA	NA	NA	NA	Yes
CTRL 3	55	M	-	-	-	-	-	NA	NA	NA	NA	NA	NA	NA	No
CTRL 4	70	M	-	-	-	-	-	NA	NA	NA	NA	NA	NA	NA	No
CTRL 5	59	M	-	-	-	-	-	NA	NA	NA	NA	NA	NA	NA	No

## Data Availability

All data needed to evaluate the conclusions are available in the main text or the Supplementary Materials.

## Supplementary Materials

The following supporting information can be downloaded at https://www.mdpi.com/article/10.3390/cells15161505/s1. Supplementary Table S1. List of the labelled internal standards used for extraction procedures. The final concentration (Conc) added is expressed as µM. In the last column, the respective lipid classes for which abundance was derived as pmol normalized to cell count; Supplementary Table S2. Primers used for the Real-time PCR analysis; Supplementary Table S3. Antibodies used in this study; Supplementary Table S4. Mean values and standard deviation (SD) of 284 lipid species amounts (pmol/106 cells) identified in control (Ctr) and treated (O-αSyn).

## Abbreviations

ACAA1: Acetyl-CoA Acyltransferase 1; ACOT: Long-chain acyl-CoA thioesterases; α-MSH: α-melanocyte-stimulating hormone; αSyn: alpha-synuclein; BCL11: B-cell lymphoma/leukemia 11; BLIMP1: B-lymphocyte-induced nuclear maturation protein 1; CYP11A1: Cytochrome P450 Family 11 Subfamily A Member 1; DEFB4B: defensin beta 4B; DEGS2: Delta(4)-desaturase, sphingolipid 2; EMA: epithelial membrane antigen; ESM1: endothelial cell-specific molecule 1; ETC: electron transport chain; FABP: fatty acid-binding protein; FADS2: fatty acid desaturase 2; FDPS: Farnesyl Diphosphate Synthase; FAs: Free fatty acids; GBA: Glucocerebrosidase; GCMS: Gas Chromatography–Mass Spectrometry; GPAM: Glycerol-3-phosphate acyltransferase; HexCers: Hexosylceramides; HILIC: Hydrophilic Interaction Liquid Chromatography; HRMS: High Resolution Mass Spectrometry; K7: keratin 7; K10: keratin 10; LCMS: Liquid Chromatography-Mass Spectrometry; LD: lipid droplet; MC-R: melanocortin receptor; MUFAs: monounsaturated fatty acids; O-αSyn: oligomeric αSyn; PD: Parkinson’s disease; PLIN2: perilipin 2; PPARγ: peroxisome proliferator-activated receptor gamma; PSAP: Prosaposin; Q-TOF: Quadrupole Time of Flight; RP-HPLC: Reversed Phase-High Performance Liquid Chromatography; SCD1: stearoyl-CoA desaturase 1; SD: seborrheic dermatitis; SERPINB: Serpin Peptidase Inhibitor, Clade B; SFAs: saturated fatty acids; SG: Sebaceous gland; SGPP1: Sphingosine-1-Phosphate Phosphatase 1; SLC52A1: Solute Carrier Family 52 Member 1; SMs: Sphingomyelins; SMPD2: Sphingomyelin Phosphodiesterase 2; SPHK1: Sphingosine Kinase; SREBP1: Sterol Response Element-Binding Protein-1; STS: Steroid Sulfatase; TGs: Triglycerides; TSLP: Thymic Stromal Lymphopoietin.
