# Supplementary material for: Effects of α-Synuclein on the Lipid Phenotype of SZ95 Human Sebocytes: A Preliminary Study in the Context of Parkinson’s Disease
Source: Cells. 2026 Aug 21;15(16):1505. doi: 10.3390/cells15161505 (PMC13510847; doi:10.3390/cells15161505)
Supplement: Supplementary file 1 [file cells-15-01505-s001.zip › cells-4484531-supplementary.pdf]

330713 ǀ Deuterated Ceramide LIPIDOMIX®					
**Compound name**	**Formula**	**Molecular weight**	**Exact mass**	**Conc µM**	**Lipid class**
C16 Ceramide-d7 (d18:1-d7/16:0)	C34H60D7NO3	544.94	544.5560	8	
C18 Ceramide-d7 (d18:1-d7/18:0)	C36H64D7NO3	573.00	572.5873	4	
C24 Ceramide-d7 (d18:1-d7/24:0)	C42H72D7NO3	657.16	652.6499	8	
C24:1 Ceramide-d7 (d18:1-d7/24:1(15Z))	C42H74D7NO3	655.14	654.6656	4	
**330731 ǀ EquiSPLASH™ LIPIDOMIX®**					
**Compound name**	**Formula**	**Molecular weight**	**Exact mass**	**Conc µM**	**Lipid class**
15:0-18:1(d7) PC	C41H73D7NO8P	753.11	752.6061	27	PCs, PCs O-
18:1(d7) Lyso PC	C26H45D7NO7P	528.72	528.3921	38	LPCs
15:0-18:1(d7) PE	C38H67D7NO8P	711.03	710.5591	28	PEs, PEs O-
18:1(d7) Lyso PE	C23H39D7NO7P	486.64	486.3451	41	
15:0-18:1(d7) PG (Na Salt)	C39H67D7NaO10P	764.02	763.5357	26	PGs
15:0-18:1(d7) PI (NH4 Salt)	C42H75D7NO13P	847.13	846.5963	24	PIs
15:0-18:1(d7) PS (Na Salt)	C39H66D7NNaO10P	777.02	776.5309	26	
15:0-18:1(d7)-15:0 TG	C51H89D7O6	812.37	811.7646	25	
15:0-18:1(d7) DG	C36H61D7O5	587.98	587.5506	34	DGs
18:1(d7) MG	C21H33D7O4	363.59	363.3366	55	
18:1(d7) Chol Ester	C45H71D7O2	658.16	657.6441	30	CEs
d18:1-18:1(d9) SM	C41H72D9N2O6P	738.12	737.6397	27	SMs
C15 Ceramide-d7 (d18:1-d7/15:0)	C33H58D7NO3	530.92	530.5404	38	
**In-house Standards MIX**					
**Compound name**	**Formula**	**Molecular weight**	**Exact mass**	**Conc µM**	**Lipid class**
Hexadecanoic-d17 Acid (d17-PA)	C16H15D17O2	273.53	273.3469	200	FFAs, FAHFAs
n-Hexadecyl-1,1,2,2-d4 Hexadecanoate-16,16,16-d3	C32H57D7O2	487.90	487.5346	200	
Glyceryl trihexadecanoate-d98 (d98TG 48:0)	C51D98O6	905.92	905.3515	100	TGs
Deuterated cholesterol sulfate sodium salt (d7-CHS)	C27H38D7NaO4S	495.34	495.3376	50	CHS
N-palmitoyl-d31-D-erythro-sphingosine (d31-Cer16:0)	C34H37D30NO3	569.10	567.7004	5	Ceramides, HexCers
Deuterated cholesterol-2,2,3,4,4,6-d6	C27H40D6O	392.70	392.3925	200	Cholesterol

Supplementary Table S1. List of the labelled internal standards used for extraction procedures. The final concentration (Conc) added is expressed as μM. In the last column, the respective lipid classes for which abundance was derived as pmol normalized to cell count.

Gene	Oligonucleotide sequences (5′-3′)	Amplicon size	Accession Number
ACAA1	F: GACAGGTCATCACGCTGCTCAA<br>R: CCAGGGTATTCAAAGACGGCAG	115 bp	NM_001607.4
ACOT2	F: ATGGAGACGCTCCATCTGGAGT<br>R: GTGATGCCCTTCAGGAAAGAGG	149 bp	NM_006821.6
ACOT6	F: GAGCAATCCACTGGAGGAACAC<br>R: GAGCTTGTAGCCTTTCAGAGGC	146 bp	NM_001037162
ACOT8	F: GCTGACCACTGGATGCTCTATG<br>R: AGGTCACAGCTAGGACTCCATC	106 bp	NM_005469
ADIPOQ	F: TGCTGATAATGACAATGAC<br>R: CTCTGAGTTAGTGGTGAT	78 bp	NM_004797.4
BCL11A	F: CTCGTTCTGCACATGGAGCTCT<br>R: GCAAGAGAAACCATGCACTGGTG	141 bp	NM_022893
BCL11B	F: CTCCCTTTGGATGCCAGTGTCA<br>R: GGCTCCAGGTAGATGCGGAAG	145 bp	NM_138576
BLIMP1	F: AAGTGTAACTCCAGCACTGTG<br>R: CCAAAACGTGTGCCCTTTGGTATG	290 bp	NM_001198.4
CA2	F: AACAATGGTCATGCTTTCAACG<br>R: TGTCCATCAAGTGAACCCCAG	128 bp	NM_000067.3
CCL26	F: GCTGCTTCCAATACAGCCACA<br>R: TCCTTGGATGGGTACAGACTTTC	135 bp	NM_001371936.1
CYP11A1	F: TGGCATCCTCTACAGACTCCTG<br>R: CTTCAGGTTGCGTGCCATCTCA	145 bp	NM_000781
DEFB4B	F: CTCAGCTCCTGGTGAAGCTC<br>R: AAGGCAGGTAACAGGATCGC	129 bp	NM_001205266.2
DEGS1	F: CTATGCGTTTGGCAGTTGCA<br>R: CAGTTGCCAAAGGCAGCATT	81 bp	NM_001321541.2
DEGS2	F: GGTGCCTGTGCCCTACATTA<br>R: GGCCTCAAGCTCCACTCATC	135 bp	NM_206918.3
DGAT1	F: TGGTGTGTGGTGATGCTGATC<br>R: GCCAGGCGCTTCTCAA	194 bp	NM_012079.6
DGAT2	F: TGGGGGCTGGTGCCCTACTC<br>R: AATTGGCCCCGAAGGCTGGC	215 bp	NM_001253891.2
ESM1	F: ACTTGCTACCGCACAGTCTCAG<br>R: AATCCATCCCGAAGGTGCCGTA	142 bp	NM_007036.5
FABP4	F: CCTGGACTGAAGTTCGCATT<br>R: TGCAAATTTCCCATTTCCTT	238 bp	NM_001442.3
FABP5	F: TGAAGGAGCTAGGAGTGGGA<br>R: TGAACCAATGCACCATCTGT	220 bp	NM_001444.3
FADS2	F: TGTCTACAGAAAACCCAAGTGG<br>R: TGTGGAAGATGTTAGGCTTGG	128 bp	NM_001281502
FAS	F: GACCGCTTCCGAGATTCC	137 bp	NM_004104

	R: GATGGCAGTCAGGCTCAC		
GAPDH	F: TGCACCACCAACTGCTTAGC<br>R: GGCATGGACTGTGGTCATGAG	198 bp	NM_001289746
GBA	F: CCTACTCATGCTGGATGACCA<br>R: CCAATGTACAGCAATGCCATGAA	106 bp	NM_00157.4
GLB1	F: CACTCCACAATCAAGACCGAAGC<br>R: CTGTGCTGCATAGGGTGAGTTG	141 bp	NM_000404.4
GLUL	F: TTGAGAAACTAAGCAAGCGG<br>R: CTACACCAGCAGAAAAGTCG	129 bp	NM_002065.6
GLUT1	F: TGGCATCAACGCTGTCTTCT<br>R: AGCCAATGGTGGCATACACA	83 bp	NM_006516.4
GLUT3	F: AGCTCTCTGGGATCAATGCTGTGT<br>R: ATGGTGGCATAGATGGGCTCTTGA	85 bp	NM_006931.3
GPAM	F: TTGTGGCTTGCCTGCTCCTCTA<br>R: AATCACGAGCCAGGACTTCCTC	102 bp	NM_020918
HK1	F: CCGACCGCCAGCATGA<br>R: ACTTGTCAATCTTTTTGACCTGG	85 bp	NM_000188.3
IL1α	F: CGCCAATGACTCAGAGGAAGA<br>R: AGGGCGTCATTCAGGATGAA	120 bp	NM_000575
IL1β	F: CTGAGCTCGCCAGTGAAATG<br>R: TTTAGGGCCATCAGCTTCAAA	77 bp	NM_000576.2
IL6	F: AGCCACTCACCTCTTCAGAACG<br>R: GGTTCAGGTTGTTTTCTGCCAG	141 bp	NM_000600.5
IL8	F: CTTGGCAGCCTTCCTGATTTC<br>R: TTCTGTGTTGGCGCAGTGTG	168 bp	NM_000584
K10	F: TGATGTGAATGTGGAAATGAATGC<br>R: GTAGTCAGTTCCTTGCTCTTTTCA	147 bp	NM_000421.5
MC5R	F: CTCAACCTGAATGCCACAGAG<br>R: GTCTGCTATCACTAGGTGCTTG	291 bp	NM_005913.3
PDK1	F: TGGAACACCATGCCAACAGA<br>R: CCTCCTCGGTCACTCATCTTC	97 bp	NM_002610.5
PGK1	F: CCGCTTTCATGTGGAGGAAGAAG<br>R: CTCTGTGAGCAGTGCCAAAAGC	149 bp	NM_000291.4
PLIN2	F: TGAGATGGCAGAGAACGGTG<br>R: GCAATTTGCGGCTCTAGCTT	90 bp	NM_001122.4
PPARγ	F: GCCAAGCTGCTCCAGAAAAT<br>R: TGATCACCTGCAGTAGCTGCA	73 bp	NM_138711
PSAP	F: GCCTCCAAGAATGTCATCCCTG<br>R: CAATCAGCTTGGTCACCTCCTTC	127 bp	NM_002778.4
SCD1	F: CATAATTCCCGACGTGGCTTT<br>R: AGGTTTGTAGTACCTCCTCTGGAACA	150 bp	NM_005063

SERPINB3	F: GAGAACACCACAGGAAAAGCTGC<br>R: TCTCCGAAGAGCTTGTTGGCGA	140 bp	NM_006919.3
SERPINB4	F: GGAGCCAAAGACAACACTGCAC<br>R: CTGGTGATGAACATTTCCTGACC	123 bp	NM_002974.4
SGPP1	F: CTGGTGTTCTCTAGTTTGCCTAAG<br>R: GGTTGAAGTTGTCAATCAGGTCC	137 bp	NM_030791.4
SIRT3	F: CCCTGGAAACTACAAGCCCAAC<br>R: GCAGAGGCAAAGGTTCCATGAG	162 bp	NM_012239
SLC52A1	F: GCAGAGCTTTTCCTGTTTGCCC<br>R: CCAGCAGAGAAAGACCAACCAG	146 bp	NM_017986
SLC7A5	F: TGTTTTCAAGCACAACCC<br>R: AATAAACATGGTGCCGACA	112 bp	NM_003486.7
SMPD2	F: TGGTGCTCAACGCCTATGTGAC<br>R: GTCTGCCTTCTTGGATGTGTGG	131 bp	NM_030791.4
SPHK1	F: GCTGGCAGCTTCCTTGAACCAT<br>R: GTGTGCAGAGACAGCAGGTTCA	126 bp	NM_182965.3
SPT	F: TGGTCATTTGGCCCAGGTC<br>R: TTCCAACCATTGGCTTCACATC	121 bp	NM_004863.4
SQLE	F: TGGTTACATGATTCATGATC<br>R: TACTGAACTCCCATCACAAC	216 bp	NM_003129.4
SREBP1	F: TTAGAGCGAGCACTGAAC<br>R: TGGAACTGATGGAGAAGC	157 bp	NM_001005291
SREBP2	F: CCGCCTGTTCCGATGTACAC<br>R: TGCACATTCAGCCAGGTTCA	56 bp	NM_004599.4
STS	F: GCTGGCAAAAGTCAACACGGAG<br>R: GTCCGATGTGAAGTAGATGAGGG	129 bp	NM_000351.7
TSLP	F: TATCTGGTGCCCAGGCTATTCG<br>R: TGAAGCGACGCCACAATCCTTG	134 bp	NM_033035

Supplementary Table S2. Primers used for the Real-time PCR analysis.

Primary antibodies				
Antigen	Code	Host	Dilution	
			Sebocytes	Skin biopsies
α-Synuclein	S3062 Merck	Rabbit	/	1:200
α-Synuclein	Syn211 (#AHB0261) Invitrogen, Thermo Fisher Scientific	Mouse	1:200	/
PLIN2/ADFP	#MA5-38601 Invitrogen, Thermo Fisher Scientific	Mouse	1:1000	1:400
GAPDH	G9545Sigma-Aldrich (Milan, Italy)	Rabbit	1:5000	/
K7	#4465 Cell Signaling (Danvers, MA, USA)	Rabbit	1:1000	/
BLIMP1	#9115 Cell Signaling (Danvers, MA, USA)	Rabbit	1:1000	/
PPARγ	D9 #2430 Cell Signaling (Danvers, MA, USA)	Rabbit	1:1000	/
PPARγ	sc-7273 Santa Cruz Biotechnology (Uithoorn, 1422 DR, The Netherlands)	Mouse	/	1:50
SREBP1	sc-13551 Santa Cruz Biotechnology (Uithoorn, 1422 DR, The Netherlands)	Mouse	1:1000	/

EMA	ab156947 Abcam (Cambridge, UK)	Mouse	1:1000	/
FADS2	ab72189 Abcam (Cambridge, UK)	Rabbit	1:1000	/
Secondary antibodies				
Fluorochrome/Enzyme Antibody	Code	Host	Dilution	
anti-mouse IgG HRP	Cell Signaling (Danvers, MA, USA)	Goat	1:3000	
anti-rabbit IgG HRP	Cell Signaling (Danvers, MA, USA)	Goat	1:8000	
Alexa Fluor® 555 anti-rabbit	Thermo Fisher	Goat	1:500	
Alexa Fluor® 488 anti-mouse	Thermo Fisher	Goat	1:500	

Supplementary Table S3. Antibodies used in this study.

	**Mean**	**Mean**	**SD**	**SD**
**pmol/10⁶ cells**	**Ctr**	**O-αSyn**	**Ctr**	**O-αSyn**
**15Me-C17:0**	1880,82	2042,69	162,949	132,248
**14Me-C17:0**	3323,05	3818,57	166,161	178,141
**C12:0**	6056,08	8206,24	797,218	2330,16
**C13:0**	877,516	1543,95	359,817	437,775
**C14:0**	24379,7	30581	1921,1	3692,82
**C15:0**	5253,98	5524,36	482,062	526,74
**C16:0**	338800	438180	49187,1	89851,4
**C17:0**	7758,47	7027,56	959,18	419,981
**C18:0**	323052	369125	29533,5	71729,9
**C20:0**	6346,84	4502,25	1513,33	778,918
**C21:0**	434,119	446,255	41,6338	15,3095
**C22:0**	2722,36	2454,12	314,617	98,2997
**C23:0**	1647,58	1694,25	112,738	52,6483
**C24:0**	2143,54	2240,5	154,658	57,5882
**C25:0**	638,576	673,516	24,1239	60,9987
**C26:0**	19030,1	23105,6	1756,98	2787,53
**C27:0**	510,298	549,834	65,858	32,4813
**C28:0**	347,14	356,334	20,4775	20,7068
**C29:0**	399,54	402,969	21,0976	28,6557
**C30:0**	195,326	211,027	11,811	14,0157
**C14:1**	999,007	1107,64	133,104	66,5965
**C15:1**	599,589	694,476	117,448	55,8903
**C16:1n10**	429,259	583,889	176,778	175,568
**C16:1n7**	34,2275	69,9994	7,3365	42,5411
**C17:1**	14426,8	20424,8	1184,32	7779,19
**C18:1**	11149,7	17558	3895,66	7226,8
**C18:1 (II)**	6595,26	9697,44	2058,21	4756,21
**C19:1**	339,736	402,82	11,1373	20,7328
**C20:1**	676,962	835,425	30,2892	69,4005
**C21:1**	106,232	116,926	7,14853	2,47817
**C22:1**	339,258	417,756	54,8389	30,9018
**C23:1**	431,123	482,225	27,2453	29,2761
**C24:1**	408,83	497,334	35,1761	47,9044
**C25:1**	104,975	107,765	7,47672	4,15443
**C26:1**	117,934	152,503	10,8035	16,3462
**C27:1**	159,332	164,383	1,50765	5,06179
**C28:1**	351,776	389,303	23,2356	15,8256
**C29:1**	245,703	234,995	13,4158	14,5298
**C30:1**	89,1178	97,0518	10,7256	8,00013
**FAHFA 36:3;O**	90,6541	111,378	12,7736	4,61471

**FAHFA 38:6;O**	191,685	205,153	8,56964	4,16191
**FAHFA 38:4;O**	198,258	259,093	26,1965	39,8651
**FAHFA 38:3;O**	1318,89	1661,08	225,15	538,412
**FAHFA 39:3;O**	216,531	264,863	46,1154	65,0907
**FAHFA 40:5;O**	113,159	155,244	15,4002	22,4259
**FAHFA 40:4;O**	485,176	719,961	87,9952	127,981
**FAHFA 41:4;O**	1256,41	2287,15	176,443	549,775
**FAHFA 42:3;O**	91695,1	103224	10069,1	14591,1
**FAHFA 42:6;O**	142,27	167,091	19,3096	17,6095
**FAHFA 42:5;O**	419,325	636,764	136,576	83,903
**FAHFA 42:4;O**	734,296	956,462	122,844	177,11
**FOH C15:0**	268,051	364,794	228,212	236,815
**FOH C16:0**	867,353	1251,71	142,522	437,979
**FOH C18:0**	7169,82	7561,28	819,861	2018,38
**FOH C18:1**	32,0725	53,0676	17,0256	9,17603
**Cholesterol**	8270,4	12147,3	1673,61	2127,51
**CHS**	69,3517	97,738	11,5181	15,5177
**TG 44:0**	9,3119	12,0839	0,71794	1,92241
**TG 45:0**	5,30835	11,2571	0,67435	2,77934
**TG 46:0**	22,5566	34,0386	2,4084	5,54826
**TG 47:0**	9,73762	7,06613	1,62306	4,59466
**TG 48:0**	58,0772	74,446	9,07192	3,77741
**TG 49:0**	7,00818	8,23055	1,45087	4,24611
**TG 50:0**	83,2603	106,112	10,2923	24,3072
**TG 51:0**	5,21342	6,62213	1,50403	1,32216
**TG 52:0**	241,406	320,912	40,8955	84,6276
**TG 54:0**	590,584	844,657	124,451	218,672
**TG 44:1**	17,1969	40,8264	3,52944	12,1678
**TG 46:1**	23,0547	35,6081	6,54397	8,10204
**TG 47:1**	6,93206	12,7804	0,57998	4,85311
**TG 48:1**	73,419	109,241	12,238	20,2492
**TG 49:1**	16,2562	14,1143	3,7109	5,19312
**TG 50:1**	232,002	347,315	40,6544	69,0396
**TG 51:1**	18,9495	23,3235	3,78642	4,70959
**TG 52:1**	202,863	298,746	31,1232	73,6518
**TG 53:1**	13,7483	17,0161	2,75568	4,49052
**TG 54:1**	63,4089	91,2301	9,75406	29,9125
**TG 56:1**	10,2417	27,0263	2,43455	9,39167
**TG 48:2**	46,9014	60,9879	13,2412	18,8853
**TG 49:2**	232,596	249,294	13,8951	22,9207
**TG 50:2**	159,249	241,991	35,7614	53,8967
**TG 51:2**	31,0023	45,1755	4,50719	12,617
**TG 52:2**	467,524	695,722	108,402	124,575

**TG 53:2**	31,761	42,7045	4,44819	9,66589
**TG 54:2**	266,742	392,408	62,6896	96,5016
**TG 55:2**	12,2715	15,7895	1,75885	4,13106
**TG 56:2**	33,7889	64,8125	6,26704	20,5109
**TG 50:3**	47,8427	69,8894	8,58485	17,281
**TG 51:3**	12,0376	17,0384	1,65345	5,40179
**TG 52:3**	149,502	227,644	35,5881	51,7831
**TG 53:3**	20,8891	33,4904	3,66357	9,19651
**TG 54:3**	274,638	442,464	50,4497	88,0743
**TG 56:3**	53,8765	84,5577	9,78298	21,3964
**TG 58:3**	13,2131	30,107	3,43908	9,65131
**TG 48:4**	124,324	192,645	27,5596	54,5836
**TG 52:4**	44,9406	61,9831	10,512	18,1194
**TG 54:4**	94,2221	154,801	20,4086	62,3947
**TG 55:4**	22,7948	31,4059	4,55543	7,62918
**TG 56:4**	54,0004	93,6778	13,1086	37,0008
**TG 58:4**	20,7586	35,9136	3,87706	13,9153
**TG 58:5**	26,6514	37,3082	5,83833	14,5641
**DG 30:0**	13,324	14,113	1,81026	2,58357
**DG 32:0**	159,397	173,832	30,4773	32,2354
**DG 33:0**	14,905	15,048	3,76773	5,08233
**DG 34:0**	2941,12	2805,31	453,806	599,199
**DG 35:0**	63,1337	51,814	14,9061	11,3183
**DG 36:0**	6450,31	6120,73	916,595	1067,83
**DG 38:0**	94,4727	66,1647	22,6295	20,6089
**DG 40:0**	61,8349	54,0038	18,3762	19,3239
**DG 30:1**	3,10815	4,72355	0,35528	1,44476
**DG 32:1**	15,9979	26,1504	9,53615	8,00611
**DG 34:1**	24,302	33,0683	10,8427	9,08267
**DG 35:1**	6,14703	6,37413	1,5491	1,1853
**DG 36:1**	57,6287	58,9397	12,4157	14,7895
**DG 32:2**	4,93061	4,85561	0,50326	0,93531
**DG 34:2**	53,7082	55,0394	6,74138	8,51101
**DG 36:2**	38,3331	44,0477	7,23658	7,57329
**DG 36:3**	20,322	20,0962	2,3723	2,5859
**DG 38:4**	13,7936	20,4903	7,28717	8,5834
**CE 18:1**	96,4646	145,178	81,211	14,4352
**CE 18:2**	62,7927	83,2932	45,6473	11,6767
**CE 20:2**	45,1657	62,7436	30,8824	8,271
**CE 20:3**	163,632	225,653	126,89	12,3234
**CE 22:3**	64,0948	107,605	45,9367	19,6072
**CE 20:4**	254,117	324,4	202,346	37,6431
**CE 22:4**	339,112	444,705	268,196	65,4432

**CE 22:5**	812,289	967,838	662,317	92,2675
**CE 24:5**	112,618	147,656	88,35	24,9026
**CE 26:5**	92,1931	122,169	72,1383	19,3892
**CE 22:6**	938,944	1088,61	787,503	177,347
**CE 24:6**	98,0172	88,4372	92,1986	18,8227
**CE 26:6**	42,6523	54,0848	32,1604	11,3648
**Cer[NDS]34:0**	3,44456	6,13673	0,28515	0,20973
**Cer[NDS]36:0**	2,72555	3,77013	0,34636	0,10767
**Cer[NDS]38:0**	5,29356	9,77707	1,13288	1,55705
**Cer[NDS]40:0**	15,279	23,9743	2,62668	7,12387
**Cer[NDS]42:0**	6,8151	9,70634	0,99446	1,03779
**Cer[NDS]44:0**	2,2457	2,41578	0,0366	0,18824
**Cer[NDS]46:0**	2,20369	2,1139	0,12245	0,02842
**Cer[NS]34:1**	63,6304	111,596	11,045	15,2114
**Cer[NS]35:1**	5,87831	6,36483	0,63651	0,39505
**Cer[NS]36:1**	10,2754	16,9379	1,89817	1,60815
**Cer[NS]37:1**	4,41308	4,22086	0,44569	0,35884
**Cer[NS]38:1**	7,81532	12,964	1,25195	1,51746
**Cer[NS]39:1**	4,05805	5,678	0,61281	0,6004
**Cer[NS]40:1**	44,3435	71,3234	8,63621	12,0406
**Cer[NS]41:1**	15,4649	21,7158	3,05012	3,272
**Cer[NS]42:1**	120,189	194,832	21,8766	32,3418
**Cer[NS]43:1**	3,88564	5,56828	0,72874	0,79309
**Cer[NS]44:1**	5,06284	8,26012	0,67497	1,0294
**Cer[NS]45:1**	0,91324	1,01595	0,05815	0,08837
**Cer[NS]46:1**	1,18498	1,27249	0,07206	0,06253
**Cer[AS]42:1**	39,6168	59,7769	8,38404	9,45198
**HexCer 40:0;O2**	6,76745	9,30812	1,02232	0,76839
**HexCer 42:0;O2**	9,65558	13,0124	1,51934	1,46423
**HexCer 34:1;O2**	96,5629	112,036	12,6283	5,90558
**HexCer 36:1;O2**	27,6535	31,8625	4,26346	11,0525
**HexCer 38:1;O2**	7,84441	9,20159	0,89977	0,77924
**HexCer 40:1;O2**	14,838	20,3581	2,34943	2,98586
**HexCer 42:1;O2**	18,2971	22,7419	2,92386	3,29959
**HexCer 42:2;O2**	12,2702	17,6325	2,10929	2,38363

**Hex2Cer 40:1;O2**	2,73073	3,65541	0,37004	0,24237
**Hex2Cer 42:1;O2**	5,71903	6,14841	0,42013	0,55674
**Hex2Cer 42:2;O2**	3,23321	3,7127	0,42453	0,31407
**Hex3Cer 40:1;O2**	5,7917	6,81986	1,05489	0,2725
**Hex3Cer 42:1;O2**	6,74238	7,11768	1,13153	0,51133
**Hex3Cer 42:2;O2**	6,73351	7,89255	1,22477	0,33418
**PC 28:0**	10,3787	14,6797	1,46688	2,90413
**PC 29:0**	13,6041	18,6946	1,71112	2,9195
**PC 30:1**	26,3435	31,0451	2,6029	3,64591
**PC 30:0**	148,369	189,151	17,9191	24,2923
**PC 31:1**	44,9053	54,309	3,96887	5,63721
**PC 32:3**	19,3017	24,5764	3,817	7,7255
**PC 32:2**	49,6561	55,7253	3,97612	3,73769
**PC 32:1**	291,983	339,231	26,5679	23,8978
**PC 32:0**	263,924	303,521	27,933	25,5159
**PC 35:2**	101,461	111,241	7,24588	6,07688
**PC 35:1**	72,1086	71,7689	2,29489	8,91876
**PC 34:4**	46,3735	48,2456	8,11397	5,62935
**PC 34:3**	94,5667	101,701	6,70651	1,15425
**PC 34:2**	227,22	250,883	17,0799	11,0011
**PC 34:1**	975,697	1085,39	73,7121	32,7739
**PC 36:6**	34,1061	30,2819	3,06237	12,175
**PC 36:5**	61,5085	54,364	1,81315	9,93309
**PC 36:4**	168,262	202,143	18,8942	35,7133
**PC 36:3**	190,234	210,738	21,687	18,8038
**PC 36:2**	500,47	579,776	46,0295	24,3749
**PC 36:1**	363,252	392,569	33,1767	19,0673
**PC 37:4**	64,8357	81,08	7,31898	3,36314
**PC 37:2**	39,1039	40,7135	2,3451	2,44421
**PC 38:7**	37,4692	41,703	6,98275	5,57122
**PC 38:6**	78,7566	87,8321	10,2299	5,81832
**PC 38:5**	241,151	273,115	36,9882	32,2672
**PC 38:4**	320,518	249,362	39,9025	136,376
**PC 38:3**	225,658	267,243	24,9211	29,797
**PC 38:2**	163,156	196,05	20,8677	22,2524
**PC 40:8**	29,7715	37,2653	7,81984	11,6719
**PC 40:7**	70,0261	85,4814	12,8214	19,5638

**PC 40:6**	88,1858	106,672	14,2441	19,3226
**PC 40:5**	93,1784	113,394	14,7263	19,9883
**PC 40:4**	42,4008	74,4003	36,6811	13,7449
**PC 40:3**	19,46	23,9733	2,71444	2,87142
**PC 40:2**	18,2602	24,2927	5,10675	3,70131
**PC 40:1**	9,47201	10,3055	4,95251	4,86405
**PC 42:10**	6,3515	8,3351	1,19326	1,96222
**PC 44:2**	5,53375	7,00915	0,95108	1,46975
**PC 42:9**	14,6321	22,9096	4,0932	5,90492
**PC 42:8**	20,8231	26,7725	4,03145	7,20093
**PC O-32:2**	7,18134	7,73021	0,99863	0,81172
**PC O-32:1**	18,695	20,7807	1,95351	2,517
**PC O-34:2**	148,981	180,545	13,7667	29,4943
**PC O-34:1**	110,465	127,453	9,1256	6,46777
**PC O-38:4**	42,9116	54,5351	7,62898	17,1895
**PC O-40:1**	4,93604	5,87922	0,67128	1,01931
**LPC 18:0/0:0**	5,57757	6,68763	1,45011	0,3431
**LPC 0:0/20:4**	5,90105	7,23749	1,56753	1,15439
**LPC 20:4/0:0**	3,27747	4,09753	0,82515	0,33109
**LPC 0:0/18:2**	1,13075	1,322	0,25187	0,08496
**LPC 18:2/0:0**	0,83418	0,96256	0,16949	0,04247
**LPC 0:0/18:1**	12,2826	13,6501	3,1343	0,97744
**LPC 18:1/0:0**	7,19325	8,59589	1,90529	0,33731
**LPC 0:0/16:1**	1,68037	1,9316	0,37031	0,08473
**LPC 16:1/0:0**	1,1493	1,36914	0,22702	0,07674
**LPC 0:0/16:0**	2,06392	2,55132	0,3849	0,17026
**LPC 16:0/0:0**	7,21199	9,20014	1,74806	0,48971
**SM 34:1;O2**	604,696	901,341	110,28	153,411
**SM 36:1;O2**	61,6142	84,6507	10,5093	14,7443
**SM 38:1;O2**	101,136	118,235	4,3284	8,21549
**SM 40:2;O2**	60,7394	80,8712	9,38193	13,8369
**SM 40:1;O2**	174,752	239,6	32,3027	42,0373
**SM 42:2;O2**	364,982	556,579	60,9134	94,6578
**SM 42:1;O2**	520,674	761,893	106,018	152,395
**SM 44:4;O2**	129,034	192,598	25,8567	45,4889
**SM 44:2;O2**	15,8999	24,9431	4,85232	5,98624
**SM 44:1;O2**	15,7709	22,6138	4,27184	4,64496
**SM 34:0;O2**	292,093	468,635	51,805	94,9534
**SM 35:1;O2**	20,8555	28,6215	4,29776	5,76693
**SM 36:0;O2**	24,3207	37,6047	3,91431	8,02096
**SM 41:1;O2**	43,3488	56,3724	8,30674	10,2627
**SM 41:2;O2**	91,8695	119,789	31,078	31,3819
**PE 32:2**	4,47783	6,32087	0,80073	0,83585

**PE 32:1**	26,0541	39,5003	4,92516	6,7258
**PE 34:3**	8,44022	11,8897	1,52149	1,54985
**PE 34:2**	78,9595	116,09	15,0687	18,2599
**PE 34:1**	249,944	375,691	49,5079	68,3512
**PE 36:4**	132,836	183,998	23,9139	26,2169
**PE 36:3**	716,933	1048,82	126,46	173,595
**PE 36:2**	569,944	842,992	103,639	133,841
**PE 36:1**	508,397	747,16	95,8069	128,824
**PE 38:5**	560,659	790,01	104,121	107,746
**PE 38:4**	1246,02	1774,45	224,413	273,56
**PE 38:3**	940,61	1325,27	169,958	220,68
**PE 40:5**	408,18	564,003	74,9433	88,4905
**PE 40:4**	258,109	363,425	46,4039	56,8907
**PE 40:3**	96,9887	136,483	16,4133	21,5317
**PE 40:2**	22,8537	33,3429	3,82207	3,24483
**PE 40:1**	10,2665	14,0319	1,72932	1,53674
**PE O-34:4**	3,52763	4,8323	0,55735	0,73129
**PE O-34:3**	16,2563	23,8393	2,96779	3,81866
**PE O-34:2**	130,344	200,853	24,7307	35,9052
**PE O-34:1**	26,4414	39,265	4,69575	7,32533
**PE O-36:5**	650,601	950,98	116,085	157,184
**PE O-36:4**	89,4819	132,23	16,0784	21,4979
**PE O-36:3**	180,904	192,876	4,20289	4,40617
**PE O-36:2**	120,378	138,64	7,53579	7,86938
**PE O-38:6**	553,496	807,339	98,6581	132,862
**PE O-38:5**	765,195	1068,63	138,44	177,08
**PE O-38:4**	109,473	157,793	19,9991	26,049
**PE O-38:3**	28,5097	31,903	2,59847	1,91072
**PE O-38:1**	1,59188	2,0129	0,05426	0,37417
**PI 36:4**	155,633	207,377	28,1646	23,0445
**PI 36:3**	95,453	133,302	16,1641	15,2555
**PI 36:2**	191,302	266,335	32,6247	30,9007
**PI 36:1**	159,914	250,26	30,7408	52,8534
**PI 38:5**	253,993	324,938	41,4661	27,4222
**PI 38:4**	855,981	1178,26	153,098	165,498
**PI 38:3**	278,837	416,381	48,5781	63,7551
**PI 38:2**	80,5878	119,265	13,0297	17,8503
**PI 40:6**	62,7773	79,6942	9,93869	9,94812
**PI 40:5**	81,4317	109,372	13,7792	14,6534
**PI 40:4**	62,2047	82,6204	9,35223	11,2282
**PG 36:1**	8,43654	12,6142	1,41108	2,388
**PG 36:2**	4,02766	5,8754	0,74892	0,74947
**PG 34:1**	25,4294	37,2136	4,71893	7,26352

Supplementary Table S4. Mean values and standard deviation (SD) of 284 lipid species amounts (pmol/106 cells) identified in control (Ctr) and treated (O-αSyn).
